# Combining Masculinizing Resistance, Rotation, and Biocontrol to Achieve Durable Suppression of the Potato Pale Cyst Nematode: A Model

**DOI:** 10.1111/eva.70012

**Published:** 2024-09-19

**Authors:** Israël Tankam Chedjou, Josselin Montarry, Sylvain Fournet, Frédéric M. Hamelin

**Affiliations:** ^1^ Institut Agro Univ Rennes, INRAE, IGEPP Rennes France

**Keywords:** biological control, crop rotation, demo‐genetic model, durable management of resistance, *Globodera pallida*, integrated pest management

## Abstract

The pale cyst nematode, *Globodera pallida*, is a pest that poses a significant threat to potato crops worldwide. The most effective chemical nematicides are toxic to nontarget organisms and are now banned. Alternative control methods are therefore required. Crop rotation and biological control methods have limitations for effectively managing nematodes. The use of genetically resistant cultivars is a promising alternative, but nematode populations evolve, and virulent mutants can break resistance after just a few years. Masculinizing resistances, preventing avirulent nematodes from producing females, might be more durable than blocking resistances, preventing infection. Our demo‐genetic model, tracking both nematode population densities and virulence allele frequencies, shows that virulence against masculinizing resistance may not be fixed in the pest population under realistic agricultural conditions. Avirulence may persist despite the uniform use of resistance. This is because avirulent male nematodes may transmit avirulent alleles to their progeny by mating with virulent females. Additionally, because avirulent nematodes do not produce females themselves, they weaken the reproductive rate of the nematode population, leading to a reduction in its density by at least 20%. This avirulence load can even lead to the collapse of the nematode population in theory. Overall, our model showed that combining masculinizing resistance, rotation, and biocontrol may achieve durable suppression of *G. pallida* in a reasonable time frame. Our work is supported by an online interactive interface allowing users (i.e., growers, plant health authorities, researchers) to test their own control combinations.

## 1 Introduction

Nematodes, that is, microscopic roundworms, are the most abundant animals on Earth (Van Den Hoogen et al. 2019). Among them, plant‐parasitic nematodes pose major threats to crops worldwide, and cyst nematodes are among the most damaging species (Orlando and Boa 2023). Potato (*Solanum tuberosum*) is currently the fourth major crop in the world and is a staple food in many regions. Potato cyst nematodes cause significant yield losses and are therefore a food security concern (Coyne et al. 2018). Costs due to yield losses and control measures reach billions of US dollars each year worldwide (Jones et al. 2013).

The Pale cyst nematode, *Globodera pallida*, is a major potato pest now present worldwide (Orlando and Boa 2023). *Globodera pallida* is a quarantine organism in most countries including Europe and North America (Dandurand et al. 2019; Price et al. 2021), which means that strict control measures must be applied upon nematode detection. These measures include restrictions on the movement of plant material, and interruption of susceptible potato cultivation until nematode density is below the detection threshold.

For decades, chemical nematicides have been used to control potato cyst nematodes. However, chemical nematicides are toxic to nontarget organisms and cause other environmental harm (Desaeger, Wram, and Zasada 2020). Over the past decade, the most effective chemical nematicides have therefore been banned. Growers must now adapt and use alternative control methods such as rotation, biocontrol, and resistance (Zasada et al. 2010; Varandas, Egas, and Conceicao 2020).

Rotation consists of growing nonhost crops instead of growing potatoes every year since *G. pallida* cannot reproduce in the absence of potatoes. However, the effectiveness of crop rotation is limited since the hatching of cyst nematode eggs is only stimulated by hatching factors exuded by host roots (Guerrieri et al. 2021; Shimizu et al. 2023), but also because eggs can survive within cysts for years in the absence of a host. Nevertheless, spontaneous hatching of *G. pallida* larvae occurs at a rate of 30% per year (Turner 1996). Larvae hatching from eggs do not survive longer than 2 weeks without a host (Robinson, Atkinson, and Perry 1987). A complementary method would thus be to use a trap crop, which is a nonhost crop, which, however, triggers hatching (e.g., Hooks et al. 2010). Trap‐crop strategies have shown effectiveness in reducing cyst nematode infestations, with studies reporting population reductions ranging from 20% to over 90% (e.g., Mhatre et al. 2021). However, only a few plant species are known as trap crops (Scholte 2000; Hickman and Dandurand 2023), and growers may be reluctant to grow species that do not provide an income (Orlando and Boa 2023).

Biocontrol includes the use of natural enemies such as fungi (Tobin et al. 2008; Contina, Dandurand, and Knudsen 2017), trap plants (Kushida et al. 2003; Dandurand, Zasada, and LaMondia 2019), or the combination of both (Dandurand and Knudsen 2016). Biocontrol agents can produce nematicidal compounds, colonize plant roots, or parasitize nematode eggs (Abd‐Elgawad 2020). However, using living organisms is much more challenging than applying a molecule, due to the complexity of ecological interactions occurring in the field (Knudsen et al. 2015). Interestingly, it has been shown that cyst nematode populations can be reduced by applying hatching factors to infested soil in the absence of a host, thereby inducing a “suicide hatch” of eggs (Devine and Jones 2000, 2001). In particular, exogenously applied root exudates can induce up to 80% hatching of *G. pallida* eggs in the absence of the host plant (Ngala et al. 2021, 2024). However, biocontrol products are often less effective in the field than under controlled conditions (Le Mire et al. 2016).

Resistance means growing genetically resistant cultivars, as opposed to genetically susceptible cultivars. Resistance can be very effective in controlling *G. pallida* (Price et al. 2023). The problem is parasite populations evolve and often break down resistance genes after a few years, whereas a breeding program may take at least a decade (Brown 2015). Breakdown of resistance by *G. pallida* populations has been reported in Germany and The Netherlands over the last decade (Niere, Krüssel, and Osmers 2014; Mwangi et al. 2019; Grenier et al. 2020). From now on, we will use the term “virulent” to denote resistance‐breaking pest genotypes.

There are two types of resistance against *G. pallida*. Blocking resistance occurs when the nematode larvae cannot achieve their development cycle on the host. Masculinizing resistance occurs when *G. pallida* larvae can infect the host, but cannot exploit it well enough to produce females. The latter are much larger than the males and transform into cysts when died. The cyst is the only structure which is transmitted from one season to the next. Avirulent nematodes can only produce males, which act only as gamete‐like propagules and do not survive long in the soil. However, avirulent males may mate with virulent females and transmit avirulent alleles to their progeny. This way, avirulence may persist in the population, despite host resistance (Schouten 1993, 1994). Because virulence may not be fixed in the population, masculinizing resistance might be more durable than blocking resistance (Schouten 1996).

Wild *Solanum species* provide major genetic resistances. Masculizing resistances derive from *S. vernei* and *S. spegazzinii*, while blocking resistance derives from *S. sparsipilum* (Mugniery et al. 2007; Fournet et al. 2013). Regarding *G. pallida*, which is diploid, virulence may be dominant (with respect to *S. spegazzinii* and *S. sparsipilum*) or recessive (w.r.t. *S. vernei*). European potato‐resistant cultivars all come from *S. vernei*, meaning that resistance is masculinizing and virulence recessive. Virulence recessiveness is expected to increase resistance durability (Saubin et al. 2021). While virulence is theoretically associated with a fitness cost, Fournet et al. (2016) found no evidence of a fitness cost associated with virulence in *G. pallida*.

To summarize, rotation, biocontrol, and resistance are effective but separately are not sufficient to durably control *G. pallida*. These control methods should therefore be used in combination. However, carrying out experiments to test all possible combinations over multiple years would be long and costly, especially since *G. pallida* is a quarantine pest.

Mathematical modeling makes it possible to simulate disease dynamics over multiple years and compare control strategies. Key biological features of *G. pallida*, such as virulence recessiveness, require the development of a model tracking both nematode population densities and genetic frequencies, that is, a demo‐genetic model. *G. pallida* has low dispersal ability and makes only one generation per year. Therefore, we used a discrete‐time spatially implicit model to explore whether combining rotation, biocontrol, and resistance can achieve durable suppression of *G. pallida* in a reasonable time frame.

## 2 Model

### 2.1 Life Cycle and Notations

**FIGURE 1:**
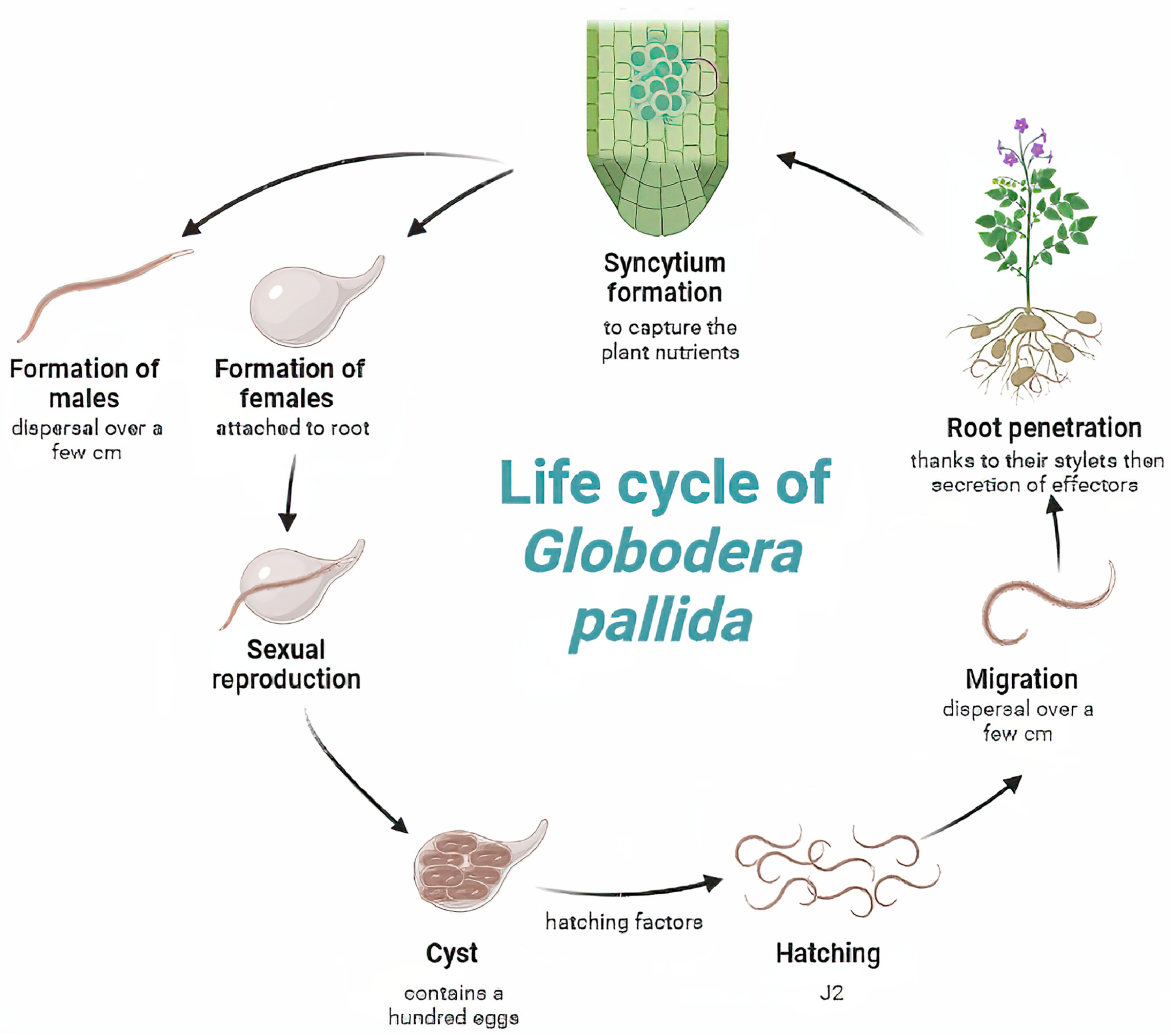
Life cycle of the pale cyst nematode *G. pallida*. The cycle begins with egg production within a protective cyst formed by the body of a female nematode (bottom), followed by the hatching of infective juveniles (J2) that seek a host, responding to root exudates. The nematode matures within the host, differentiating into males and females, with males being smaller and thinner (top left). The male mates with the female, which leads to cyst formation.

The life cycle of *G. pallida*, summarized in Figure 1 (see also Figure S1), begins with the survival stage, the cyst. The cyst, which measures approximately 0.5 mm in length, contains *e* eggs (Nicol et al. 2011). These eggs are enveloped within a protective cyst, which acts as a shield against environmental stresses and chemical treatments. Encysted eggs have a mortality fraction *μ*, meaning that a fraction 1 − *μ* of them survive each year as long as they are protected by a cyst (Turner 1996).

When the eggs hatch, they release juvenile nematodes, called second‐stage juveniles (J2). These J2 are nonfeeding and possess a vermiform morphology, allowing them to move actively through the soil in search of a host plant. The J2 are motile and exhibit chemotaxis, gravitating towards root exudates and other cues emitted by potential host plants. A fraction *s* of them survive until they find a suitable root (Ewing, Blok, and Kettle 2021).

After locating a suitable host, the larva enters the host root cells using its highly specialized stylet, a piercing mouthpart. This marks the onset of parasitism, as *G. pallida* establishes a permanent feeding site within the host root releasing chemicals that induce the formation of a syncytium that supplies nutrients to the nematode (Jones and Northcote 1972).

The larva then differentiates into either a male, with probability *m*, or a female, with probability 1 − *m*. Males are smaller and thinner than females and play a role in sexual reproduction. Females become sedentary, continuing to feed on host root tissues and growing in size.

As the eggs accumulate within the female body, the female gradually enlarges and becomes more distinct in shape and color. The mature dead female, now filled with eggs, becomes a hardened structure, the cyst, capable of surviving harsh conditions in the soil. However, an average fraction *h* of cysts accidentally hatch under unsuitable conditions like the lack of a host, leading the larvae to die prematurely (Turner 1996). The cysts can remain dormant for several years, awaiting the presence of a suitable host plant to initiate a new cycle of infection.

### 2.2 Parameter Estimation

Parameters such as the survival fraction of hatched larvae, (*s*), the number of eggs per cyst, (*e*), and the male fraction, (*m*), were directly obtained from the literature (Table 1). More specifically, *s* = 0.25 is the survival fraction under optimal environmental conditions for the nematode (Ewing, Blok, and Kettle 2021), *e* = 500 is the maximum observed egg number per cyst (Nicol et al. 2011; Contina, Dandurand, and Knudsen 2019), and *m* = 0.35 is the maximum observed male fraction (Fournet et al. 2013). Our default parameter values are therefore favorable to the nematode population dynamics and relatively conservative from a control perspective. For instance, Ward, Rabbinge, and Den Ouden (1985) report a number of eggs per cyst which is half of that which we considered. We detail below how we estimated the yearly mortality of encysted eggs, (*μ*), and the yearly fraction of accidental hatching, (*h*).

**TABLE 1:** Parameter meanings and their default values.

Par.	Meaning	Default value	References
*s*	Survival fraction of larvae	0.25	Ewing, Blok, and Kettle (2021)
*e*	Number of eggs per cyst	500	Nicol et al. (2011)
*m*	Male fraction in the progeny	0.35	Fournet et al. (2013)
*μ*	Yearly egg mortality fraction	0.10	Turner (1996)
*h*	Yearly accidental hatching fraction	0.17	Turner (1996)
*b*	Biocontrol efficacy fraction	0–1	Variable
τ	Acceptance threshold	1 per g	Moxnes and Hausken (2007)
*R*	Reproduction number of *G. pallida*	65	$R=e\left(1-\mu \right)\left(1-h\right)s$
*c*	Intraspecific competition parameter	0.4 g	Section 2.4

#### 2.2.1 Estimating the Yearly Egg Mortality Fraction (*μ*)

Eggs can survive in cysts for several years, but their survival fraction declines over the years. It has been estimated that after 13 years of potato absence, about 73.4% of encysted eggs died (Turner 1996). Assuming mortality is constant, the yearly survival fraction can be estimated as ${\left(1-0.734\right)}^{\frac{1}{13}}=0.9$, which is consistent with later studies (Christoforou et al. 2014; Contina, Dandurand, and Knudsen 2019). We thus estimate the egg mortality fraction, *μ*, is about 10%.

#### 2.2.2 Estimating the Yearly Fraction of Accidental Hatching (*h*)

A decline of 91% of cysts was observed after 13 years of potato absence (Turner 1996). Assuming accidental hatching is a constant, the yearly fraction of accidental hatching can be estimated as $1-{\left(1-0.91\right)}^{\frac{1}{13}}\approx 0.17$. This fraction is comparable to, but more conservative than, the accidental hatching fraction due to water in a controlled environment, which is about 25%–30% (Gautier et al. 2020; Ngala et al. 2021). It might happen that controlled conditions more frequently trigger accidental hatching than field conditions, which would explain the difference.

Table 1 lists parameter meanings and reference values.

### 2.3 Basic Reproduction Number

Reproduction numbers are epidemiological metrics quantifying the potential for pathogens and parasites to spread. Several factors compose *G. pallida*'s reproduction number *R*, which is the number of secondary infections generated by a single female, in a susceptible host population, at low nematode density, and in the absence of control. The first one is the number of eggs, *e*, produced by a single female during her reproductive lifespan. The others are the viability of eggs inside the cyst, 1 − *μ*, the fraction of eggs that survives accidental hatching in‐between seasons, 1 − *h*, and the survival fraction of larvae in the soil, *s*:

(1) $$R=e\left(1-\mu \right)\left(1-h\right)s.$$

Using Equation (1) and the parameter values in Table 1, we obtain R≈65.

Multiplying the reproduction number, *R*, by the proportion of females, (1 − *m*), yields the *basic* reproduction number *R*(1 − *m*), that is the average number of daughters generated by a single mother, in a susceptible host population, and at low nematode density. Therefore, *G. pallida* is expected to spread and persist if $R\left(1-m\right)>1$, or to go extinct if $R\left(1-m\right)\leq 1$. Using parameter values in Table 1, we obtain $R\left(1-m\right)\approx 42$, which is much greater than 1, as expected in the absence of control.

The mathematical derivation of these reproduction numbers is provided in Supplementary Material S1.3.

### 2.4 Basic Demographic Model

From now on, *G. pallida* population density is expressed in nematode eggs per gram of soil, as in earlier models (Ward, Rabbinge, and Den Ouden 1985; Phillips, Hackett, and Trudgill 1991; Ewing, Blok, and Kettle 2021).

**FIGURE 2:**
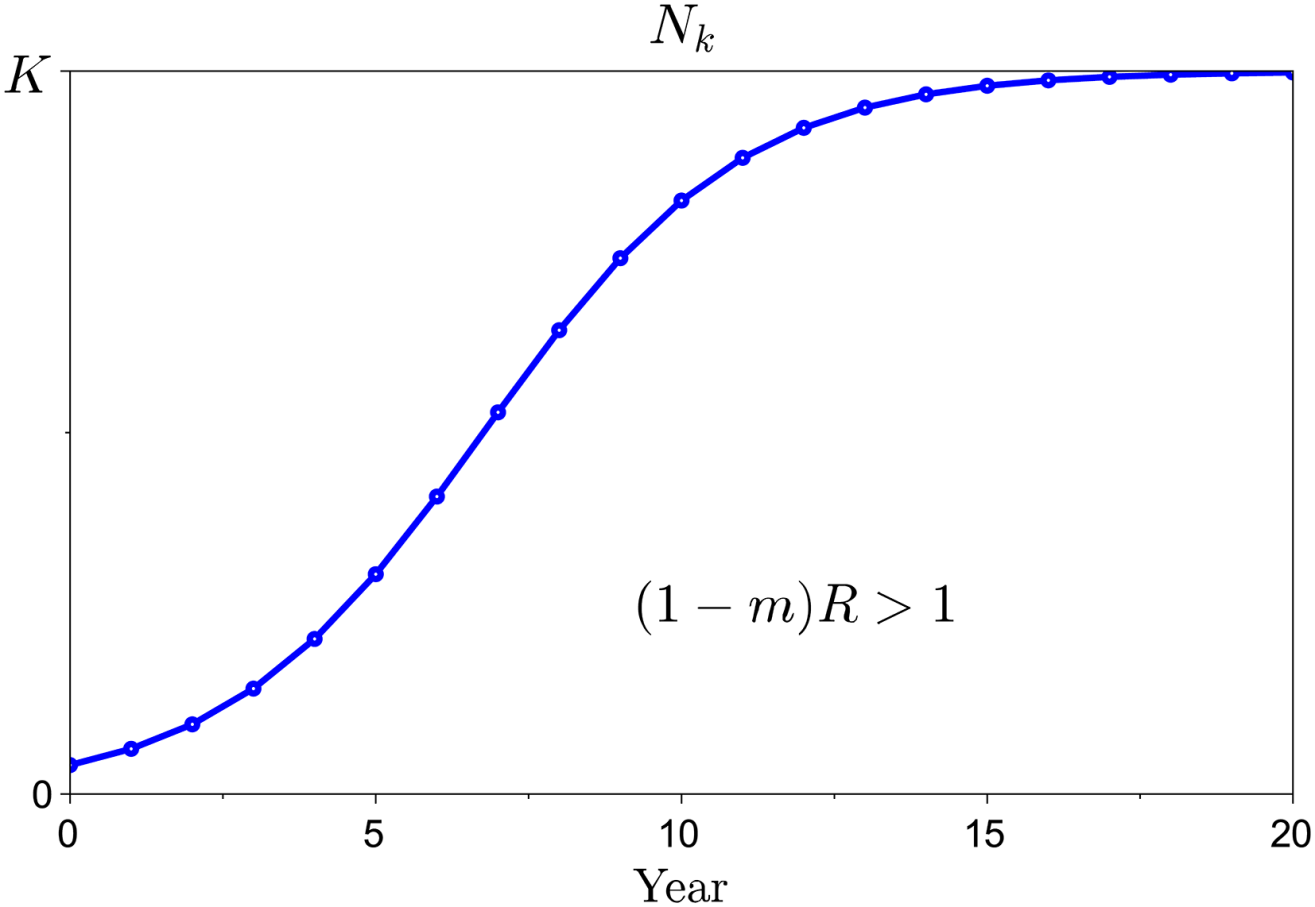
Nematode population growth in a fully susceptible (meaning not genetically resistant) host population. In the absence of resistance, the nematode population grows until it reaches the carrying capacity *K*, provided the basic reproduction number of *G. pallida* is greater than unity, that is, $\left(1-m\right)R>1$.

In a susceptible host population and in the absence of control, the nematode population grows logistically over the years. The discrete‐time analog of the continuous‐time logistic growth equation is the Beverton–Holt model (De Vries et al. 2006). Mathematical models used in the plant nematology literature often have a Beverton–Holt form, for example, Jones and Perry (1978, eq. 6) and Phillips, Hackett, and Trudgill (1991, eq. 4). Let *N*_*k*_ be the nematode population density at the beginning of year *k* (that is the number of cysts times the number of eggs per cyst). Beverton–Holt growth can be expressed as follows: for all k=0,1,2,…,

(2) $$N_{k+1}=\left(1-m\right)R\frac{N_{k}}{1+{\mathrm{cN}}_{k}},$$

in which c>0 is an intraspecific competition parameter. The term $\left(1-m\right)R$ is the basic reproduction number of the parasite. It is the average number of female nematodes that a single female produces for the next generation. If $\left(1-m\right)R>1$, the parasite population grows until it reaches carrying capacity

(3) $$K\left(R,m,c\right)=\frac{\left(1-m\right)R-1}{c},$$

as shown in Figure 2. Otherwise (if $\left(1-m\right)R<1$), the parasite population decreases to zero.

As an order of magnitude, and using parameter values in Table 1, one may consider $K\left(65,0.35,c\right)=100$ eggs per gram of soil, for example, Ward, Rabbinge, and Den Ouden (1985, fig. 2) and Jones and Perry (1978, fig. 8). Using Equation (3), we estimate $c=\left(\left(1-0.35\right)65-1\right)/100\approx 0.4\;g$.

### 2.5 Demo‐Genetic Models

We present now the effects of blocking and masculinizing resistances on the nematode population dynamics.

#### 2.5.1 Virulence Dynamics With Blocking Resistance

Blocking resistance refers to the inability of avirulent larvae to complete their development cycle and mature into adults (Mugniery et al. 2007). Consequently, they fail to reproduce. Only virulent nematodes can reproduce and therefore contribute to the overall offspring production. The exclusive selection of virulent nematodes for reproduction results in the fixation of virulence from the second generation. This enables us to model the variation of the virulence frequency $v_{k}$ simply as follows:

(4) $$\left\{\begin{array}{c} v_{0}=v_{0},\\ v_{k}=1,\;\text{for}\;k\geq 1 \end{array}.\right.$$

#### 2.5.2 Virulence Dynamics With Masculinizing Resistance

Masculinizing resistance means avirulent larvae can enter roots but fail to establish good‐quality feeding sites, resulting in differentiation into males only. However, virulent larvae are somehow able to bypass resistance and establish good feeding conditions in resistant hosts. Virulent larvae thus differentiate into males with probability *m* and into females with probability $\left(1-m\right)$.

The gene locus for virulence is diallelic: avirulence (*A*) is dominant, while virulence (*a*) is recessive. Therefore, the *Aa* and *AA* genotypes express the avirulent phenotype, whereas the *aa* genotype expresses the virulent phenotype.

We assume homogeneous mixing and random mating (panmixia) of male and female nematodes. We also assume that in the presence of the host, all cysts hatch. Therefore, the nematode generations do not overlap. This enables us to model the dynamics of the virulence frequency $v_{k}$ (that is the frequency of the virulent genotype *aa*) as:

(5) $$v_{k+1}=\frac{2\times {\mathrm{mv}}_{k}N_{k}+1\times \left(1-v_{k}\right)N_{k}}{2\times {\mathrm{mv}}_{k}N_{k}+2\times \left(1-v_{k}\right)N_{k}}=\frac{{\mathrm{mv}}_{k}+\frac{1}{2}\left(1-v_{k}\right)}{{\mathrm{mv}}_{k}+\left(1-v_{k}\right)}.$$

The above fraction is the frequency of the virulence allele (*a*) in the male progeny. Virulent larvae which differentiate into males, ${\mathrm{mv}}_{k}N_{k}$, are all homozygous (*aa*), and therefore have two virulence alleles (*a*) each. Avirulent larvae, $\left(1-v_{k}\right)N_{k}$, all differentiate into males, are all heterozygous (*Aa*), and therefore have one virulence allele (*a*) each. The frequency of the virulence allele (*a*) in the male population drives the genotypic frequency of virulence (*aa*) since all females are homozygous (*aa*). The mathematical derivation of the model is provided in more detail in Supplementary Material S1.3.

#### 2.5.3 Demo‐Genetic Dynamics

On year *k*, the fraction of larvae that can differentiate into females and therefore produce cysts is proportional to the virulence frequency $v_{k}$. The demographic model (2) thus becomes:

(6) $$N_{k+1}=\left(1-m\right)R\frac{N_{k}}{1+{\mathrm{cN}}_{k}}v_{k}.$$

Since we assume there is no cost of virulence, a monomorphic population of virulent nematodes grows as if the host were susceptible, that is, following Equation (2). Thus, the demo‐genetic model incorporating blocking resistance aligns with the basic demographic model starting from the second generation onward.

For these reasons, the remainder of the study will focus on the demo‐genetic model with masculinizing resistance. We rescale the demographic dynamics (6) by introducing $n_{k}={\mathrm{cN}}_{k}$. Combined with the genetic dynamics (5), it forms the following demo‐genetic model, tracking both population densities and gene frequencies:

(7) $$\left\{\begin{array}{l} n_{k+1}=\left(1-m\right)R\frac{n_{k}}{1+n_{k}}v_{k},\\ v_{k+1}=\frac{{\mathrm{mv}}_{k}+\frac{1}{2}\left(1-v_{k}\right)}{{\mathrm{mv}}_{k}+\left(1-v_{k}\right)} \end{array}.\right.$$

We note that Model (7) depends on two parameters only, *R* and *m*. We also note that model (7) holds for masculinizing resistance only.

### 2.6 Additional Control Methods

Additional control methods considered in this study are biocontrol and rotation. We will explore whether the combination of the two methods, together with resistance, can achieve effective and lasting suppression of *G. pallida*.

As the potato growing season is relatively short on an annual scale (16–18 weeks), we assume, for simplicity, that annual cyst mortality is the same regardless of whether the host absence period is 1 year or less (1 year minus the growing season).

We model rotations as regular potato cultivation breaks of r years, meaning that the potato is grown once every r+1 years. We consider that alternative crops to potatoes (or the absence of crops) have the same effect on nematodes (no trap crop is used). We will refer to the parameter r as the “rotation number.”

We model the biocontrol efficacy as the percentage of nematodes that do not survive biocontrol application, *b*. We assume that biocontrol is applied every year, regardless of whether potatoes are grown or not.

Under these assumptions, taking the two control methods into account simply amounts to updating the reproduction number (1) as:

(8) $$R^{\prime }=e{\left[\left(1-\mu \right)\left(1-h\right)\left(1-b\right)\right]}^{r+1}s,$$

in which the term in square brackets is the annual survival fraction of eggs, due either to natural mortality within cysts (*μ*), accidental hatching (*h*), or hatching induced by biological control (*b*). Egg survival is reduced exponentially as the rotation number (*r*) increases since mortality factors have a multiplicative effect. The demo‐genetic model (7) remains unchanged, except that *k* is the number of generations rather than the number of years passed, which becomes $\left(r+1\right)k$.

## 3 Results

### 3.1 Virulence Dynamics

Equation (7) shows that the virulence (*v*_*k*_) dynamics are independent of the nematode population dynamics (*N*_*k*_). They only depend on the male fraction in the virulent progeny, *m*. There are two possible outcomes, as detailed in Supplementary Material S1.3:

- If m≥0.5, then $v_{k}\to 1$ as k→∞: virulence ultimately fixes in the nematode population.
- If m<0.5, then $v_{k}\to v^{⋆}<1$: virulent and avirulent phenotypes are bound to coexist. The frequency of the virulent genotype is

(9) $$v^{⋆}=\frac{1}{2\left(1-m\right)}.$$

**FIGURE 3:**
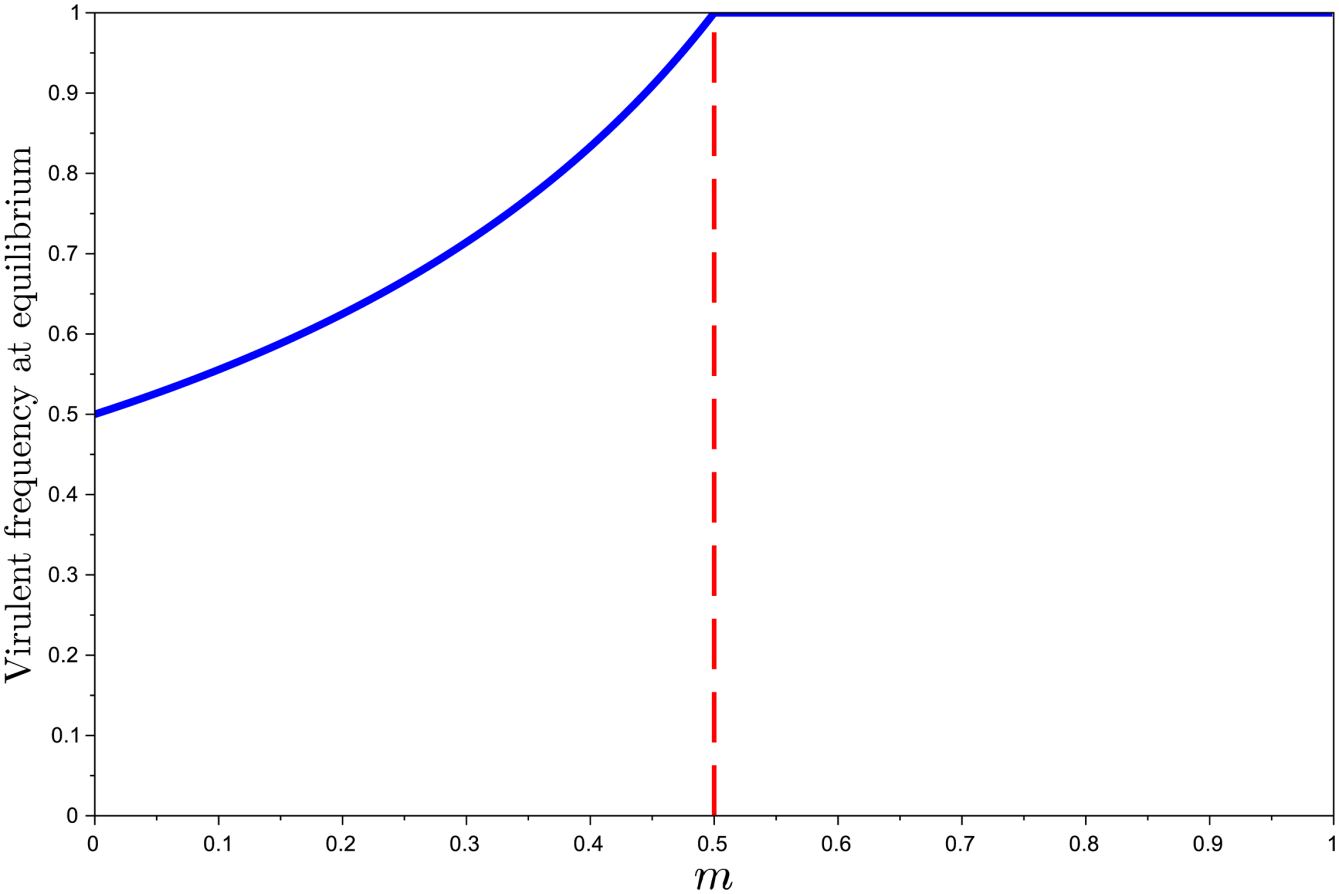
Frequency of the virulent genotype (*aa*) at equilibrium, $v^{⋆}$, as a function of the male fraction in the virulent progeny, *m*. For m≥0.5, virulence fixes in the population. For m<0.5, $v^{⋆}$ is given by Equation (9).

The second case $\left(m<0.5\right)$ is termed “incomplete selection for virulence” in Schouten (1993). Figure 3 shows $v^{⋆}$ as a function of *m*. We recognize (Schouten 1993, fig. 5), which derives here from a demo‐genetic model. Incomplete selection for virulence only occurs when the male fraction in the virulent progeny, *m*, is lower than one‐half. In this case, virulent males are not frequent enough to prevent avirulent males from effectively transmitting their genetic material by mating with virulent females. When mated with an avirulent male, females can produce both virulent (homozygous) and avirulent (heterozygous) offspring. This allows avirulent genes to persist in the population through a phenomenon akin to genetic hitch‐hiking: avirulent genetic material “hitches a ride” on virulent females. By contrast, if the male fraction of the virulent progeny exceeded 0.5, virulent nematodes would produce a sufficient fraction of males to effectively prevent avirulence from persisting through hitch‐hiking, because the fraction of heterozygotes would be critically lower in the female progeny. Specifically, the fraction of avirulent genetic material passed on would decrease over the years, ultimately leading to virulence fixation. Note that the model accounts for polyandry, meaning that one female can mate with several males. So virulence fixation would not be due to mating limitation, but instead to the dilution of the avirulence allele in the progeny.

### 3.2 Masculinizing Resistance Can Theoretically Suppress *G. pallida*

**FIGURE 4:**
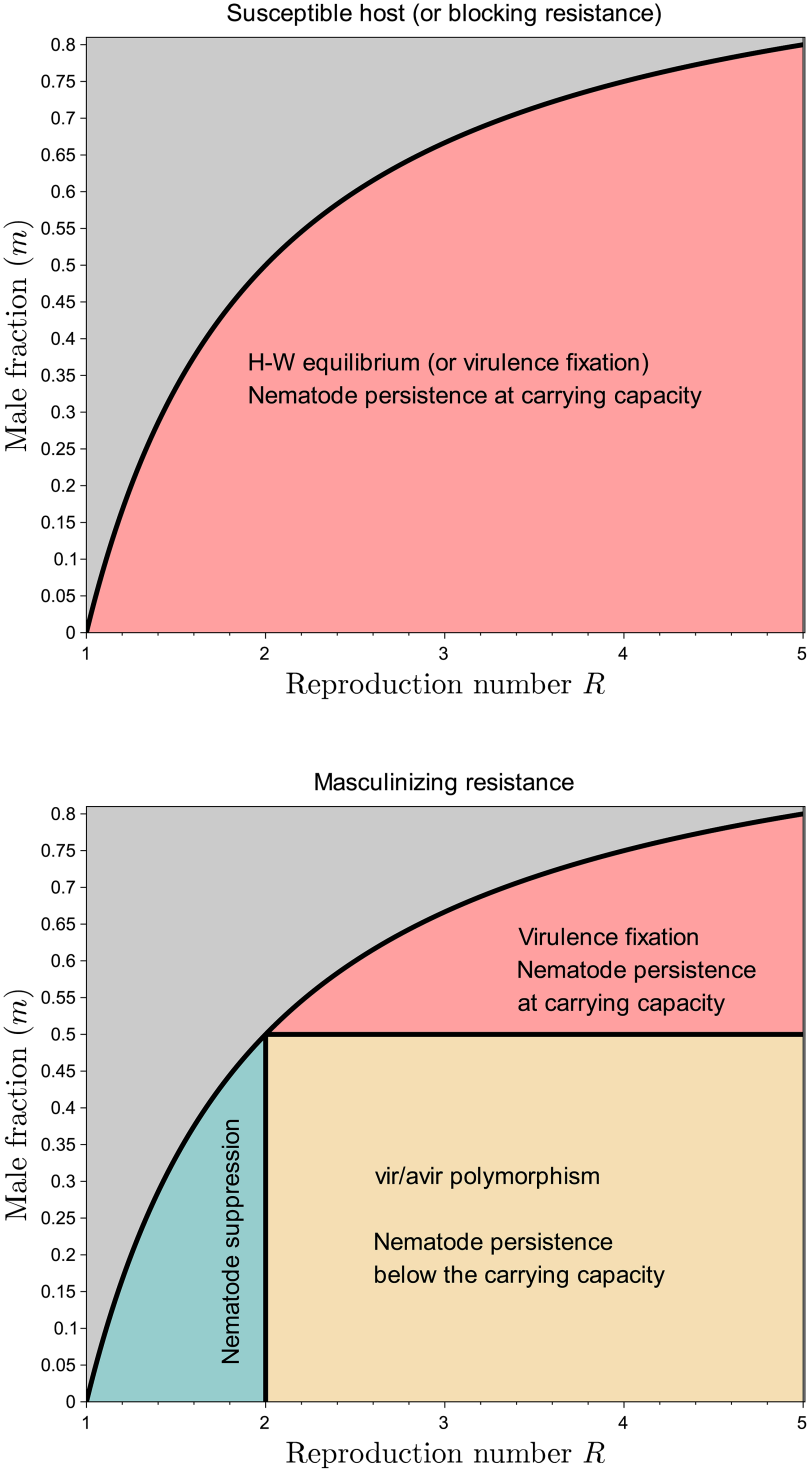
Graphical summary of the four possible outcomes of the demo‐genetic model, depending on parameter values. The model has only two (composite) parameters: The reproduction number of the parasite, *R*, and the male fraction in the virulent progeny, *m*. In case a susceptible host or a blocking resistance is grown, either the nematode population dies out (if $R\left(1-m\right)<1$; gray region) or it persists and reaches its carrying capacity (if $R\left(1-m\right)>1$; red region). In case a masculinizing resistance is grown, the picture is richer. If $R\left(1-m\right)>1$ and R<2 (green region), the nematodes go to extinction (as in the gray region), which contrasts with blocking resistance. Moreover, if R>2 and m<0.5, the nematodes persist but do not reach their carrying capacity. This is because avirulent males hitch‐hike on virulent females, as explained in Section 3.3, and proven in Supplementary Material S1.3.

Model (7) combines the virulence dynamics with the nematode population dynamics, which, as summarized in Figure 4, results in four possible outcomes, depending on parameter values (*R* and *m*). If m≥0.5, virulence fixes in the nematode population, which reaches its carrying capacity *K*. This case is however not the most relevant biologically, since m≤0.35 (Fournet et al. 2013).

More relevantly and interestingly, if m<0.5, the virulent and avirulent genotypes coexist. Two cases are again to be distinguished. If R>2, then nematodes do not reach their carrying capacity ($n_{k}\to n^{⋆}=R\left(1-m\right)v^{⋆}-1<\mathrm{cK}$, as k→∞). We will come back to this result in more detail in the next section. Otherwise, if R<2, then nematodes go to extinction ($n_{k}\to 0$ as k→∞). This means that masculinizing resistance can theoretically lead an otherwise viable nematode population to extinction. This is due to the previously mentioned avirulent male hitch‐hikers, which sort of “parasitize” the virulent population. However, the actual value of *R* is likely much greater than 2, as indicated in Table 1, in which *R* = 65 as a default value. This means that suppression, while theoretically possible, is actually implausible in the absence of additional control methods.

### 3.3 If Suppression Is Not Possible, Masculinizing Resistance May Nevertheless Decrease *G. pallida* Density by at Least 23%

**FIGURE 5:**
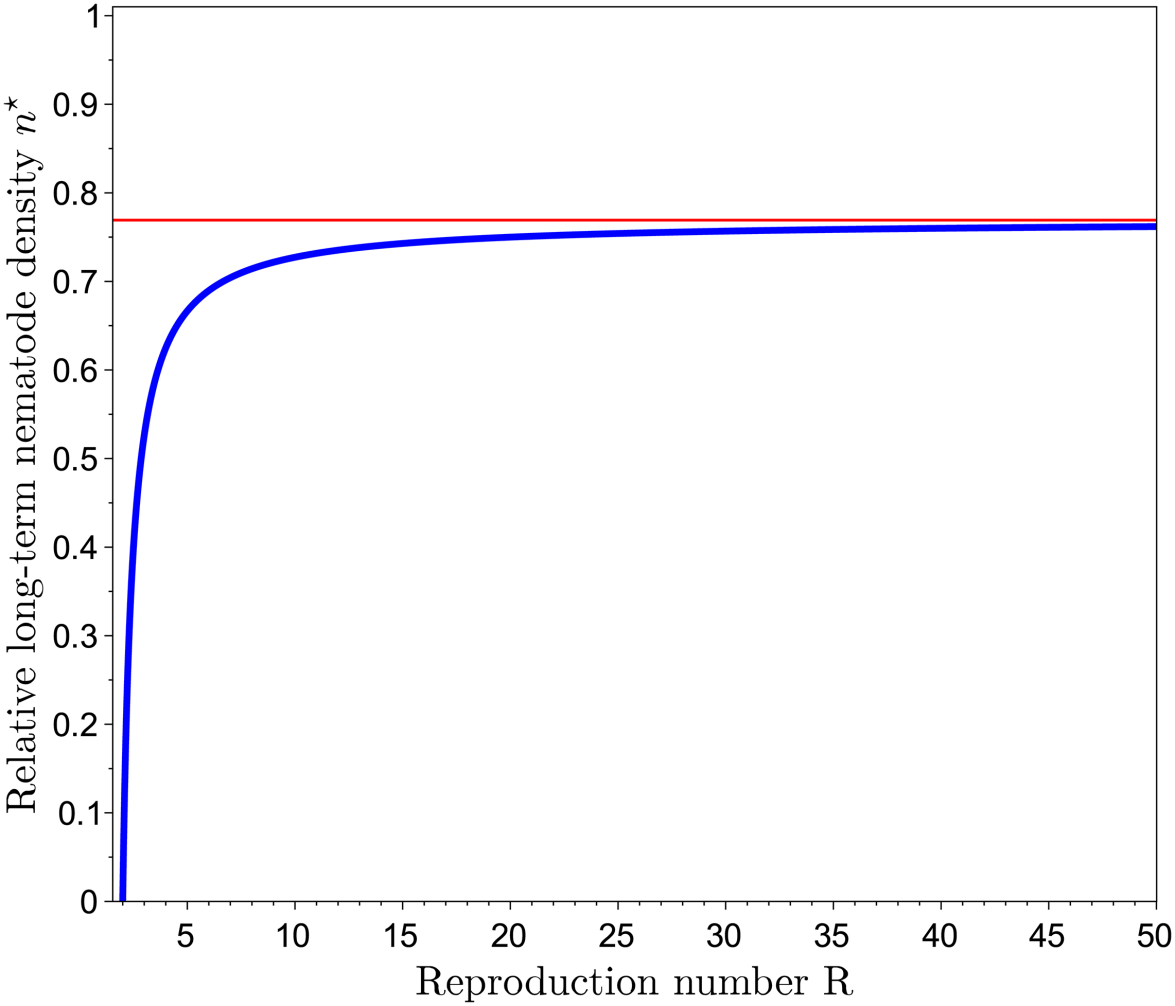
Equilibrium nematode population density (relative to carrying capacity), $\eta^{⋆}$, as a function of the reproduction number R, when growing masculinizing resistance. Parameter value: m=0.35. The red line shows the asymptotic value, $\lim_{R\to \infty }\;\eta^{⋆}=77\%$, which shows that masculinizing resistance decreases nematode density by at least 23% relative to blocking resistance or a susceptible cultivar.

Considering m=0.35<0.5, either R<2 and the nematodes are expected to die out, or R>2 and at least virulence should not fix in the population. Using Equation (9) with m=0.35, the long‐term virulence frequency is $v^{⋆}=0.77$ (Figure 3). In this case, the equilibrium nematode density (relative to carrying capacity) is

(10) $$\eta^{⋆}=\frac{n^{⋆}}{\mathrm{cK}\left(R,m,c\right)}=\frac{v^{⋆}R\left(1-m\right)-1}{R\left(1-m\right)-1},$$

as derived in Supplementary Material S1.3. Figure 5 shows that $\eta^{⋆}$ is always lower than its asymptotic value, that is, $\lim_{R\to \infty }\;\eta^{⋆}=v^{⋆}=0.77$. This result means that masculinizing resistance decreases nematode density by at least 23% compared to blocking resistance, or equivalently to a susceptible cultivar.

### 3.4 Combining Masculinizing Resistance, Biocontrol and Rotation May Achieve Long‐Term Suppression of *G. pallida* in Practice

Figure 4 shows that long‐term suppression of *G. pallida* would be possible if *R* was lower than 2 (since m<0.5). However, as indicated earlier, R≈65≫2, meaning that masculinizing resistance alone is far from being sufficient to suppress *G. pallida* in the long run.

We now consider adding biocontrol. To achieve long‐term suppression of *G. pallida*, the reproduction number taking into account biocontrol, $R^{\prime }$, defined in Equation (8), must be lower than 2. In the absence of rotation (*r* = 0), using Equations (1) and (8), the required condition is $R^{\prime }=R\left(1-b\right)<2$. This means that the biocontrol efficacy fraction, *b*, must be greater than 1−2/R to achieve long‐term suppression. Taking *R* = 65 yields b>0.97, which is not achievable in practice, since biocontrol is hardly more effective than 0.8, even under laboratory‐controlled conditions (Ngala et al. 2021; Gautier et al. 2020). Again, masculizing resistance and biocontrol alone are not sufficient to suppress *G. pallida* in the long run.

We now introduce rotations (*r* > 0). Given a biocontrol efficacy fraction *b*, the minimum rotation number *r*_min_ required for long‐term suppression is obtained from solving, using Equation (8), $R^{\prime }<2\Leftrightarrow r>r_{\min}$:

(11) $$r_{\min}=\left\lceil \frac{\log\left[\frac{2}{R\left(1-b\right)}\right]}{\log\left[\left(1-\mu \right)\left(1-h\right)\left(1-b\right)\right]}\right\rceil ,$$

in which *R* is given by Equation (1), and ⌈ ⌉ is the ceiling function.

**FIGURE 6:**
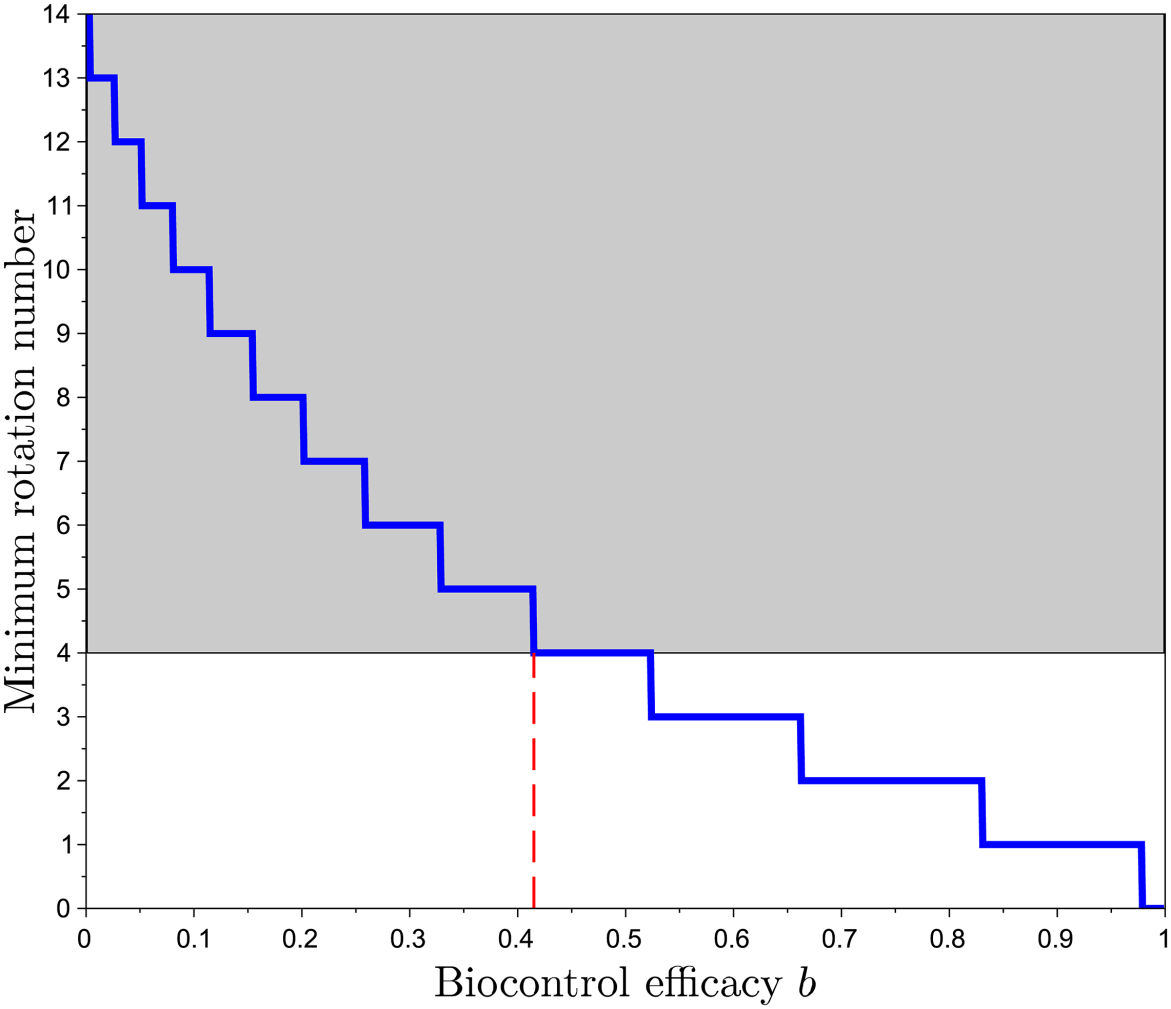
Minimum rotation number *r*_min_ required to suppress *G. pallida* in the long run as a function of the biocontrol efficacy fraction, *b* (blue lines) and default parameter values (Table 1). The rotation number should not exceed 4 in practice (white regions). The minimum biocontrol efficacy required to satisfy this constraint is indicated by the dashed red lines. The latter shows that a moderate biocontrol efficacy fraction (about 0.41), would suffice to suppress *G. pallida* in the long run. Therefore, combining masculinizing resistance, rotation, and biocontrol may achieve long‐term suppression of *G. pallida*.

Figure 6 shows, using Equation (11), the minimum rotation number required to suppress *G. pallida* in the long run, *r*_min_, as a function of the biocontrol efficacy fraction, *b* (see also Figure S2). According to practitioners, while it might be acceptable to grow potatoes every 5 years to prevent nematode infestation, this practice becomes hardly acceptable for greater rotation numbers. This means the rotation number *r* should not exceed 4 years. Not using biocontrol is equivalent to considering b=0. In the absence of biocontrol (b=0), *r*_min_ is much greater than 4. By contrast, a moderate biocontrol efficacy fraction, *b* about 0.41, would suffice to suppress *G. pallida* in the long run. Therefore, combining masculinizing resistance, rotation, and biocontrol may achieve long‐term suppression of *G. pallida*.

### 3.5 Suppressing *G. pallida* Is Achievable in a Reasonable Time‐Frame

So far we have shown that *G. pallida* can be suppressed in the long term, meaning that its population density tends to zero as time goes to infinity. In practice, however, *G. pallida* can be considered as effectively suppressed if its density does not exceed a certain acceptance threshold, τ, say 1 nematode egg per gram of soil (Moxnes and Hausken 2007). We now explore the length of time required to achieve effective suppression.

We assume m<0.5, since it is the most biologically relevant case regarding *G. pallida*. We first focus on masculinizing resistance. For simplicity, we consider that the frequency of the virulent genotype, $v_{k}$, is initially at equilibrium: that is, for all k≥0, $v_{k}=v^{⋆}$, as defined in Equation (9). Model (6) simplifies as:

(12) $$N_{k+1}=\left(1-m\right)R\frac{N_{k}}{1+{\mathrm{cN}}_{k}}v^{⋆}.$$

We next focus on dynamics leading *G. pallida* to extinction, which occurs if and only if $\left(1-m\right){\mathrm{Rv}}^{⋆}<1$, or equivalently R<2 (Figure 4). We assume $N_{0}>\tau$ (the nematode population density is initially above the acceptance threshold).

Using the explicit solution of Equation (12), that is

$$N_{k}=\frac{\left(1-\frac{R}{2}\right)N_{0}}{\left(1-\frac{R}{2}+{\mathrm{cN}}_{0}\right){\left(\frac{R}{2}\right)}^{-k}-{\mathrm{cN}}_{0}},$$

we derive the generation $k^{†}$ from which $N_{k}<\tau$ for all $k\geq k^{†}$ (Supplementary Material S1.4):

(13) $$k^{†}=\left\lceil \log\left(\frac{\left(1-\frac{R}{2}\right)\frac{N_{0}}{\tau }+{\mathrm{cN}}_{0}}{1-\frac{R}{2}+{\mathrm{cN}}_{0}}\right)\log\left(\frac{2}{R}\right)\right\rceil .$$

One can derive a similar expression when growing a susceptible host cultivar or a blocking resistance: just replace $v^{⋆}$ with 1 in the above. Note that $k^{†}$ is an upper bound under masculinizing or blocking resistance, since the virulent fraction, $v_{k}$, may take time to approach $v^{⋆}$ or 1, respectively, as k increases. Note also that, to take into account the possible use of additional control methods (biocontrol and/or rotation), R must be substituted by $R^{\prime }$, defined in Equation (8), in the above expression of $k^{†}$.

**FIGURE 7:**
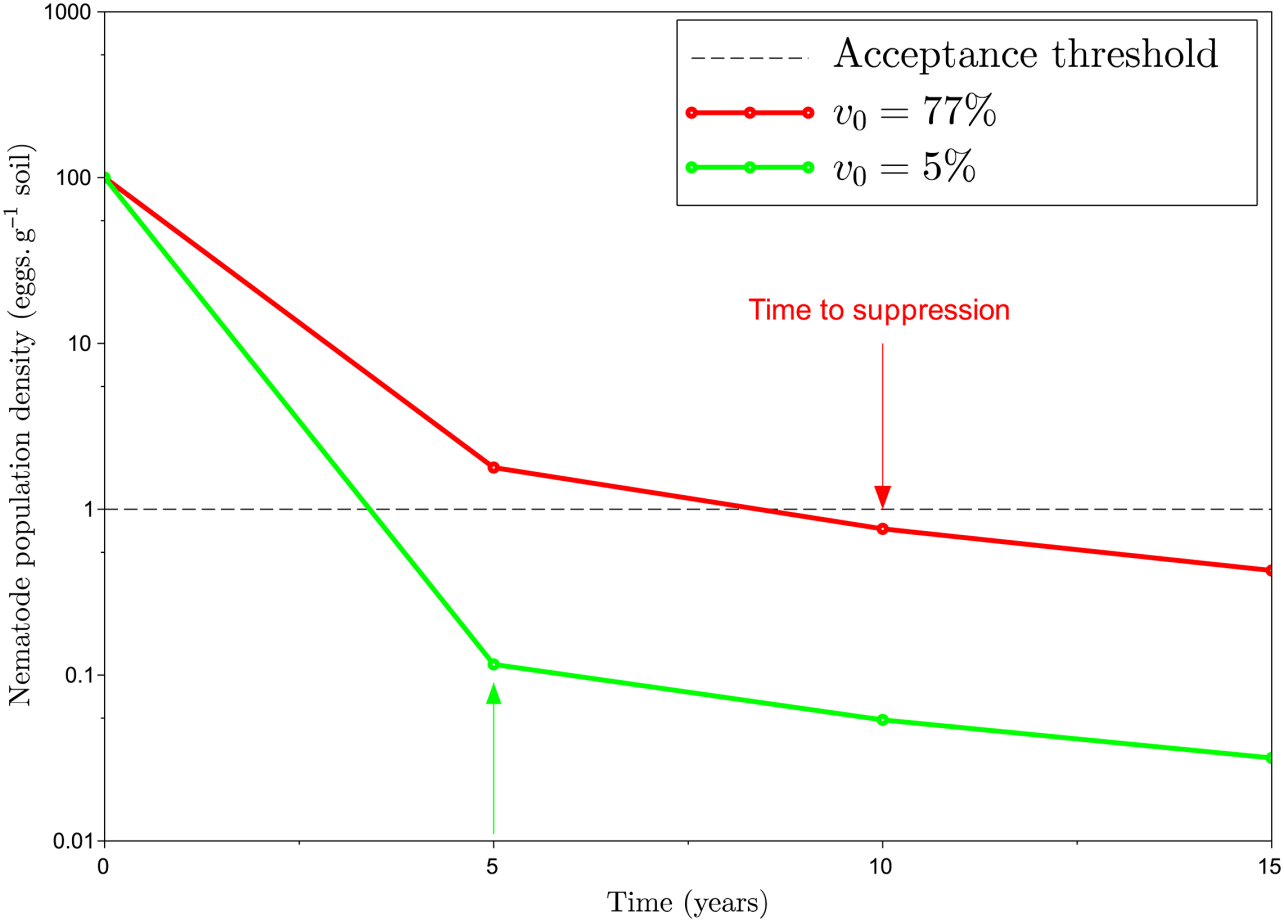
Time required to decrease nematode density under the acceptance threshold τ=1 nematode per gram of soil (time to effective suppression), with masculinizing resistance, biocontrol efficacy fraction b=0.45, rotations (r=4), and default parameter values (Table 1). Nematodes are effectively suppressed after 10 years if the initial virulence frequency is at equilibrium $v^{⋆}=1/\left(2\left(1-m\right)\right)=0.77$. For lower initial frequencies, for example, $v_{0}=0.05$, it may even take less than 5 years.

The time to suppression, $k^{†}\left(r+1\right)$, can vary significantly according to the initial nematode frequency. Consider, for instance, an initial density of 100 nematode eggs per gram of soil ($N_{0}=100$), 5‐year long rotations (r=4), and a biocontrol efficacy equal to 45% (b=0.45). Using Equation (8), this yields $R^{\prime }=1.46$, which leads to suppression of *G. pallida* since $R^{\prime }\left(1-m\right)=0.95<1$ (Figure 4). Figure 7 shows the time to effective nematode suppression with masculinizing resistance, for two different initial virulence frequencies $v_{0}$, one of which being the equilibrium frequency (*v*_0_ = v*) (see also Figure S3). According to Equation (13), it takes $k^{†}=2$ generations (i.e., $k^{†}\left(r+1\right)=10$ years) for effective nematode suppression when the initial virulence frequency $v_{0}$ is at equilibrium, versus 1 generation (5 years) with $v_{0}=5\%$ for example.

**FIGURE 8:**
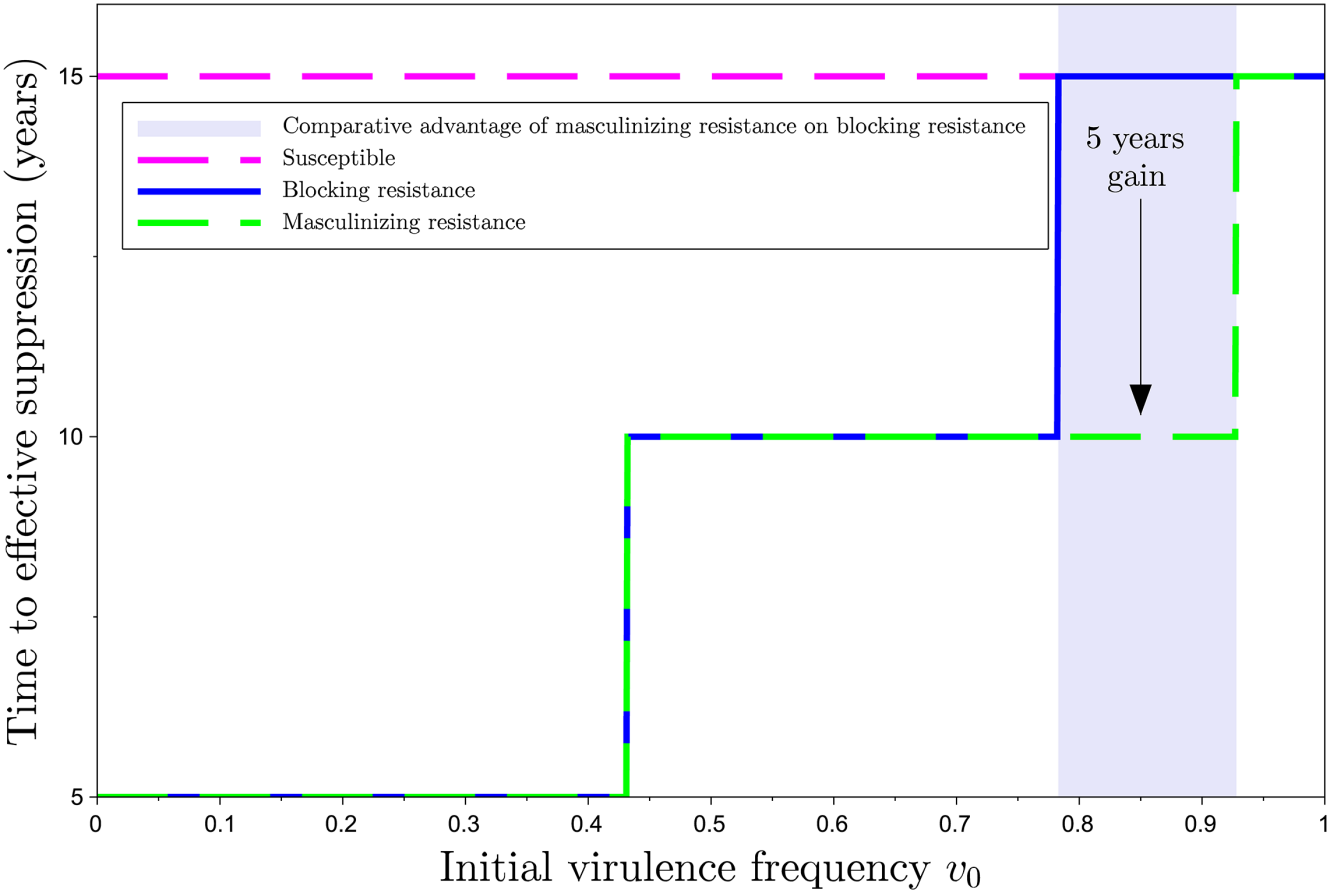
Time to effective suppression as a function of the initial virulence frequency $v_{0}$, for a biocontrol efficacy fraction b=0.45, a rotation number r=4, and default parameter values (Table 1). With these control parameter values, $R^{\prime }\left(1-m\right)=0.95<1$ meaning that the long‐term suppression of nematodes is achieved in any case. Growing a susceptible cultivar means the time to effective suppression is 15 years, regardless of the initial virulence frequency $v_{0}$. By contrast, growing a resistant cultivar means the time to effective suppression increases with the initial virulence frequency $v_{0}$. Growing masculinizing versus blocking resistance is advantageous for relatively high initial virulence frequencies ($0.8<v_{0}<0.9$), with a 5 years gain in this case.

Blocking resistance significantly decreases the nematode population in the first generation, making them more vulnerable to other control methods. This shortens the time to effective suppression compared to a susceptible cultivar (Figure 8). Blocking and masculinizing resistances perform equally when the initial virulence frequency is low enough. However, for relatively high initial virulence frequencies, blocking resistance becomes quickly ineffective for reducing nematode population densities. As a result, masculinizing resistance is a faster control method when the initial nematode frequency is relatively high (Figure 8).

The time to effective suppression under various scenarios combining biocontrol, rotation, and resistance, can be tested through our online freely available application:https://pcn‐model‐simulation.streamlit.app/


## 4 Discussion

In this study, we explored the pale cyst nematode demo‐genetics, with a particular focus on the influence of masculinizing resistance, and its interplay with biocontrol and rotation, as methods to control the potato pale cyst nematode *G. pallida*.

### 4.1 Masculinizing Resistance Can Help Control *G. pallida*

A key insight of our study is the way masculinizing resistance can help control *G. pallida*. Masculinizing resistance, as opposed to blocking resistance, can prevent virulence fixation in the nematode population by promoting the coexistence of virulent and avirulent nematode genotypes. Whether virulence fixes in the population or not is solely determined by the male fraction (m), which is the average proportion of virulent larvae that differentiate into adult males. If m exceeded 50%, virulence would be expected to fix in the nematode population. However, since m is actually lower than 50%, avirulent males can effectively transmit their genetic material by mating with virulent females. This phenomenon is akin to genetic hitch‐hiking, in that avirulent genetic material “hitches a ride” on virulent females. Consequently, virulence does not fix: its maximum frequency at equilibrium is approximately 77%. Incomplete selection for virulence was already found in a strictly genetic model (Schouten 1993, 1994), not accounting for nematode demographics.

Our model, which additionally tracks nematode population densities, originally shows that incomplete selection for virulence can theoretically lead to suppression of the nematode population. This is because avirulent larvae differentiate only into males, which do not survive host absence, unlike females, which become cysts and are the only way for nematodes to survive periods of host absence. Therefore, avirulent nematodes, which transmit their genetic material by mating with females but do not contribute to population growth, act as passengers in the nematode population. In some cases, this avirulence burden can even lead the population to extinction. However, for complete suppression to happen, the nematode reproductive number must be critically low (R<2, Figure 4). If full suppression cannot be achieved (R>2), partial suppression can nevertheless occur: the nematode population density is reduced by at least 23% (Figure 5). The presence of avirulent nematodes contributes to diluting the virulence in the nematode population, preventing nematodes from reaching their carrying capacity.

Altogether, our results show that masculinizing resistance, which maintains a genetically diverse nematode population that can be taken advantage of, may help suppress *G. pallida* in the long run. Masculinizing resistance, by reducing the damage inflicted by *G. pallida* on potato crops, therefore provides a significant advantage over blocking resistance.

### 4.2 Combining Control Methods to Achieve Suppression

Masculinizing resistance can be combined with other control measures, such as biocontrol and rotation. Our findings underscore the need to integrate multiple control methods to achieve effective nematode management. While masculinizing resistance plays a key role in preventing virulence fixation, suppression of *G. pallida* can hardly be achieved without combination with other control methods. Similarly, while biocontrol can be quite effective in reducing nematode population sizes, the biocontrol efficacy needed to achieve nematode suppression can hardly be attained without rotations. Besides, while rotations are widely used as a control method against *G. pallida* (Trudgill, Phillips, and Elliott 2014), decline rates of nematode populations in the absence of hosts may be as low as about 10% in a given year (Turner 1996). Consequently, it can take 25 years or more for cysts to be depleted of viable eggs (LaMondia and Brodie 1986; Turner 1996; Scholte 2000). Rotations should therefore be combined with other control methods. Combining biocontrol and rotation without using resistance has been experimentally addressed in (e.g., López‐Lima et al. 2013; Dandurand and Knudsen 2016). Our theoretical framework includes resistance, allows one to test flexible combinations of the three control methods, and provides information regarding the effective time frame for nematode suppression.

Our results suggest that combining masculinizing resistance, moderately efficient biocontrol, and a reasonable rotation number, can achieve nematode suppression in the long run (Figure 6). Moreover, masculinizing resistance accelerates the suppression process, as compared to blocking resistance or susceptible cultivars. However, this is heavily contingent on the initial frequency of virulent individuals (Figure 8). Masculinizing resistance confers an advantage over blocking resistance for relatively high initial frequencies of virulent nematodes. By contrast, for relatively low initial virulence frequencies, masculinizing resistance may not offer a quantitative advantage over blocking resistance in terms of suppression speed. Nonetheless, it limits maximum population size in the absence of suppression.

One novelty of this model lies in its ability to assess the efficacy of specific combinations of control methods. By setting a maximum number of rotations, we can ascertain the required biocontrol efficacy for long‐term suppression. Similarly, by determining biocontrol efficacy, we can determine the optimal number of rotations. Moreover, measuring the initial nematode density enables us to calculate the maximum time to suppression with a given strategy. Furthermore, should a tool be capable of accurately measuring the initial frequency of virulent nematodes, our model would facilitate a more precise estimation of suppression time. These results have practical implications for researchers, growers, and plant health authorities. While it might be smart to alternately use blocking and masculinizing resistances over time, testing this hypothesis goes out of the scope of the present study and is left for future research.

### 4.3 A Conservative, Worst‐Case Study

Before closing this study, we should stress that we considered the most advantageous conditions for *G. pallida*, thus representing the worst case from the grower's standpoint. Several key parameters were deliberately set to their optimal values from the nematode perspective, thereby amplifying the challenges faced in managing this agricultural pest.

First, we considered the highest possible survival rate achievable by *G. pallida*, *s* = 25% (Ewing, Blok, and Kettle 2021) and the highest possible number of eggs produced per female, *e* = 500 (Nicol et al. 2011). Second, we considered the highest possible male fraction, *m* = 35% (Fournet et al. 2013). Sexual differentiation is actually dynamic and density‐dependent. Males act as dispersal forms and are produced to a greater extent when local conditions are unfavorable to the nematode (due to, e.g., intraspecific competition or plant resistance) (Evans, Trudgill, and Brown 1977). At low nematode population density, the male fraction can be close to zero, while *m* can reach 35% for the highest population densities. Thus, in practice, *m* is likely lower than 35% in average. Therefore, avirulent nematodes, which produce only males, likely have greater access to virulent females than accounted for in the model. Thus, the burden of avirulence is likely greater in reality than in the model. The latter may therefore underestimate the suppressive effect of masculinizing resistance.

Additionally, when computing the time to effective suppression, we assumed virulence against masculinizing resistance was at its equilibrium frequency, for simplicity. However, converging to this equilibrium takes time. During the transient, virulence frequency can be lower, resulting in a reduced nematode density. Therefore, the model likely overestimates the time necessary to achieve suppression with masculinizing resistance. In practice, effective suppression might occur sooner than our model predicts. In particular, using our online application, one can check that if the initial infestation level or the initial frequency of virulence is low enough, then masculinizing resistance alone can suppress *G. pallida* in a single generation. This observation indicates that masculinizing resistance can have a particularly significant impact at the start of an outbreak.

However, we assumed the carrying capacity of the nematode population to be 100 eggs per gram of soil, which is a reasonable but somewhat arbitrary value. The actual carrying capacity likely depends on the potato cultivar considered. Cultivars hosting a larger nematode density than that considered in this study might challenge our results to some extent. Therefore, future research should explore the variability in carrying capacities among potato cultivars. This would improve the accuracy of management recommendations.

### 4.4 Genetic Drift and Its Possible Implications

Potato cyst nematodes have a small effective population size, making them particularly susceptible to the effects of genetic drift (Montarry et al. 2019). Inbreeding can further strengthen the impact of genetic drift. Inbreeding is promoted by polyandry, a mating system in which females mate with multiple males. In nematode populations, inbreeding can result in increased homozygosity for virulence alleles, making virulence more vulnerable again to genetic drift. However, our model does not yet account for genetic drift, which might impact our results to some extent.

For instance, if the male fraction *m* was greater than 50%, selection for virulence would be much stronger than genetic drift. Therefore, virulence would be expected to be fixed in the nematode population. However, in *G. pallida*, *m* is smaller than 50%, and virulence is not expected to fix in the drift‐free model. In this case, genetic drift might change the outcome of the model. Further exploration of the genetic drift dynamics in nematode populations will make the model more accurate, especially in the case of varying selection pressures and population sizes (as could happen when alternating masculinizing and blocking resistances, for instance). This exploration is left for future research.

### 4.5 Avenues for Future Research

To sum up, our study provides insight into the interplay between masculinizing resistance and nematode demo‐genetics. A key finding is that masculinizing resistance, although it alone does not guarantee nematode suppression, plays a crucial role in reducing nematode populations. Overall, our research highlights the potential of an integrated approach, combining masculinizing resistance with biocontrol and rotation, for effective long‐term control of *G. pallida*.

Looking ahead, there are promising avenues to explore further. The first is to introduce stochasticity and inbreeding effects, which will allow us to take genetic drift into account and improve the accuracy of our predictions for real‐world situations. Additionally, investigating complex crop rotation strategies alternating between resistant cultivars, susceptible cultivars, and nonhost plants, could extend the durability of nematode control. However, to promote the practical application of integrated management strategies, several key aspects should additionally be taken into account.

Genomics can provide a better understanding of the genetic bases of virulence, thus allowing the development of a molecular tool to measure the initial frequency of virulence in nematode populations. The latter will be a key input to the model and the online application.

Economic considerations play a key role in the adoption of integrated management strategies. Assessing the cost‐effectiveness of masculinizing resistance, biocontrol, and crop rotation compared to straightforward methods (e.g., using blocking resistance only) will be essential to demonstrate that integrated approaches not only help control quarantine pests but are also economically advantageous to growers.

Global variations of control strategies should also be considered. Cyst nematode management practices may vary across regions due to differences in climate, potato cultivars, and nematode genotypes. Adapting management strategies to various geographical areas, and considering the diverse needs of growers worldwide, will require specific attention. For instance, the remaining chemical nematicides authorized vary across regions and over time, as novel modes of action are getting discovered (Schleker et al. 2022). Our model explored alternative measures to chemical nematicides and therefore does not consider their specific mode of action. Such additions to the model are left for future research.

Climate change is a factor likely to have an impact on the life cycle of nematodes. For instance, as climate changes, *G. pallida*, which is currently univoltine (one generation per year), might become multivoltine (several generations per year), like other cyst nematode species (e.g., *Heterodera schachtii* or *H. carotae*). Understanding the implications of climate change on the resilience of nematodes is essential to controlling them in the long term.

## Conflicts of Interest

The authors declare no conflicts of interest.

## Supporting information


^Data S1^

## Data Availability

Data sharing is not applicable to this article as no new data were created or analyzed in this study.

## References

[eva70012-bib-0001] Abd‐Elgawad, M. M. 2020. “Biological Control Agents in the Integrated Nematode Management of Potato in Egypt.” Egyptian Journal of Biological Pest Control 30: 1–13.

[eva70012-bib-0002] Brown, J. K. 2015. “Durable Resistance of Crops to Disease: A Darwinian Perspective.” Annual Review of Phytopathology 53: 513–539. 10.1146/annurev-phyto-102313-045914 26077539

[eva70012-bib-0003] Christoforou, M. , I. S. Pantelides , L. Kanetis , N. Ioannou , and D. Tsaltas . 2014. “Rapid Detection and Quantification of Viable Potato Cyst Nematodes Using qPCR in Combination With Propidium Monoazide.” Plant Pathology 63, no. 5: 1185–1192.

[eva70012-bib-0004] Contina, J. , L. Dandurand , and G. Knudsen . 2017. “Use of gfp‐Tagged *Trichoderma harzianum* as a Tool to Study the Biological Control of the Potato Cyst Nematode *Globodera pallida* .” Applied Soil Ecology 115: 31–37.

[eva70012-bib-0005] Contina, J. B. , L. M. Dandurand , and G. R. Knudsen . 2019. “A Predictive Risk Model Analysis of the Potato Cyst Nematode *Globodera pallida* in Idaho.” Plant Disease 103, no. 12: 3117–3128. 31634034 10.1094/PDIS-04-19-0717-RE

[eva70012-bib-0006] Coyne, D. L. , L. Cortada , J. J. Dalzell , et al. 2018. “Plant‐Parasitic Nematodes and Food Security in Sub‐Saharan Africa.” Annual Review of Phytopathology 56: 381–403. 10.1146/annurev-phyto-080417-045833 PMC7340484 29958072

[eva70012-bib-0007] Dandurand, L. , I. Zasada , and J. LaMondia . 2019. “Effect of the Trap Crop, *Solanum sisymbriifolium*, on *Globodera pallida*, *Globodera tabacum*, and *Globodera ellingtonae* .” Journal of Nematology 51, no. 1: 1–11. 10.21307/jofnem-2019-030 PMC6930959 31132006

[eva70012-bib-0008] Dandurand, L.‐M. , and G. Knudsen . 2016. “Effect of the Trap Crop *Solanum sisymbriifolium* and Two Biocontrol Fungi on Reproduction of the Potato Cyst Nematode, *Globodera pallida* .” Annals of Applied Biology 169, no. 2: 180–189.

[eva70012-bib-0009] Dandurand, L.‐M. , I. A. Zasada , X. Wang , et al. 2019. “Current Status of Potato Cyst Nematodes in North America.” Annual Review of Phytopathology 57: 117–133. 10.1146/annurev-phyto-082718-100254 31100997

[eva70012-bib-0010] De Vries, G. , T. Hillen , M. Lewis , J. Müller , and B. Schönfisch . 2006. A Course in Mathematical Biology: Quantitative Modeling With Mathematical and Computational Methods. Philadelphia: SIAM.

[eva70012-bib-0011] Desaeger, J. , C. Wram , and I. Zasada . 2020. “New Reduced‐Risk Agricultural Nematicides‐Rationale and Review.” Journal of Nematology 52, no. 1: 1–16. 10.21307/jofnem-2020-091 PMC8015323 33829179

[eva70012-bib-0012] Devine, K. , and P. Jones . 2000. “Response of *Globodera rostochiensis* to Exogenously Applied Hatching Factors in Soil.” Annals of Applied Biology 137, no. 1: 21–29.

[eva70012-bib-0013] Devine, K. J. , and P. W. Jones . 2001. “Effects of Hatching Factors on Potato Cyst Nematode Hatch and In‐Egg Mortality in Soil and In Vitro.” Nematology 3, no. 1: 65–74.

[eva70012-bib-0014] Evans, K. , D. L. Trudgill , and N. J. Brown . 1977. “Effects of Potato Cyst‐Nematodes on Potato Plants V.” Nematologica 23, no. 2: 153–164.

[eva70012-bib-0015] Ewing, D. A. , V. Blok , and H. Kettle . 2021. “A Process‐Based, Stage‐Structured Model of Potato Cyst Nematode Population Dynamics: Effects of Temperature and Resistance.” Journal of Theoretical Biology 522: 110701. 33794290 10.1016/j.jtbi.2021.110701

[eva70012-bib-0016] Fournet, S. , D. Eoche‐Bosy , L. Renault , F. M. Hamelin , and J. Montarry . 2016. “Adaptation to Resistant Hosts Increases Fitness on Susceptible Hosts in the Plant Parasitic Nematode *Globodera pallida* .” Ecology and Evolution 6, no. 8: 2559–2568. 27066239 10.1002/ece3.2079 PMC4797161

[eva70012-bib-0017] Fournet, S. , M.‐C. Kerlan , L. Renault , J.‐P. Dantec , C. Rouaux , and J. Montarry . 2013. “Selection of Nematodes by Resistant Plants Has Implications for Local Adaptation and Cross‐Virulence.” Plant Pathology 62, no. 1: 184–193.

[eva70012-bib-0018] Gautier, C. , L. Martinez , S. Fournet , et al. 2020. “Hatching of *Globodera pallida* Induced by Root Exudates Is Not Influenced by Soil Microbiota Composition.” Frontiers in Microbiology 11: 536932. 33133028 10.3389/fmicb.2020.536932 PMC7578397

[eva70012-bib-0019] Grenier, E. , S. Kiewnick , G. Smant , et al. 2020. “Monitoring and Tackling Genetic Selection in the Potato Cyst Nematode *Globodera pallida* .” EFSA Supporting Publications 17, no. 6: 1874E.

[eva70012-bib-0020] Guerrieri, A. , K. Floková , L. E. Vlaar , et al. 2021. “UPLC‐MS/MS Analysis and Biological Activity of the Potato Cyst Nematode Hatching Stimulant, Solanoeclepin A, in the Root Exudate of *Solanum* spp.” Planta 254: 1–13. 34727239 10.1007/s00425-021-03766-2 PMC8563560

[eva70012-bib-0021] Hickman, P. , and L.‐M. Dandurand . 2023. “Evaluation of Solanaceous Species as Nonhost Trap Crops for *Globodera pallida* .” Journal of Nematology 55, no. 1: 20230036. 39254096 10.2478/jofnem-2023-0036 PMC10152465

[eva70012-bib-0022] Hooks, C. R. , K.‐H. Wang , A. Ploeg , and R. McSorley . 2010. “Using Marigold (*Tagetes* spp.) as a Cover Crop to Protect Crops From Plant‐Parasitic Nematodes.” Applied Soil Ecology 46, no. 3: 307–320.

[eva70012-bib-0023] Jones, F. G. W. , and J. N. Perry . 1978. “Modelling Populations of Cyst‐Nematodes (Nematoda: Heteroderidae).” Journal of Applied Ecology 15: 349–371.

[eva70012-bib-0024] Jones, J. T. , A. Haegeman , E. G. Danchin , et al. 2013. “Top 10 Plant‐Parasitic Nematodes in Molecular Plant Pathology.” Molecular Plant Pathology 14, no. 9: 946–961. 23809086 10.1111/mpp.12057 PMC6638764

[eva70012-bib-0025] Jones, M. G. K. , and D. H. Northcote . 1972. “Nematode‐Induced Syncytium—A Multinucleate Transfer Cell.” Journal of Cell Science 10, no. 3: 789–809. 5038416 10.1242/jcs.10.3.789

[eva70012-bib-0026] Knudsen, G. , L. Dandurand , J. Contina , et al. 2015. “Modeling Trap Crop and Biocontrol Agent Effectiveness in Management Strategies for *Globodera pallida* .” Aspects of Applied Biology 130: 65–74.

[eva70012-bib-0027] Kushida, A. , N. Suwa , Y. Ueda , and Y. Momota . 2003. “Effects of *Crotalaria juncea* and *C. spectabilis* on Hatching and Population Density of the Soybean Cyst Nematode, *Heterodera glycines* (Tylenchida: Heteroderidae).” Applied Entomology and Zoology 38, no. 3: 393–399.

[eva70012-bib-0028] LaMondia, J. , and B. Brodie . 1986. “The Effect of Potato Trap Crops and Fallow on Decline of *Globodera rostochiensis* .” Annals of Applied Biology 108, no. 2: 347–352.

[eva70012-bib-0029] Le Mire, G. , M. Nguyen , B. Fassotte , et al. 2016. “Review: Implementing Plant Biostimulants and Biocontrol Strategies in the Agroecological Management of Cultivated Ecosystems.” Biotechnology, Agronomy, Society and Environment 20: 299–313.

[eva70012-bib-0030] López‐Lima, D. , P. Sánchez‐Nava , G. Carrión , and A. E. Núñez‐Sánchez . 2013. “89% Reduction of a Potato Cyst Nematode Population Using Biological Control and Rotation.” Agronomy for Sustainable Development 33: 425–431.

[eva70012-bib-0031] Mhatre, P. H. , K. Divya , E. Venkatasalam , et al. 2021. “Evaluation of Trap Crop, *Solanum sisymbriifolium* and Antagonistic Crops Against Potato Cyst Nematodes, *Globodera* spp.” South African Journal of Botany 138: 242–248.

[eva70012-bib-0032] Montarry, J. , S. Bardou‐Valette , R. Mabon , et al. 2019. “Exploring the Causes of Small Effective Population Sizes in Cyst Nematodes Using Artificial *Globodera pallida* Populations.” Proceedings of the Royal Society B 286, no. 1894: 20182359. 30963865 10.1098/rspb.2018.2359 PMC6367184

[eva70012-bib-0033] Moxnes, J. F. , and K. Hausken . 2007. “The Population Dynamics of Potato Cyst Nematodes.” Ecological Modelling 207, no. 2: 339–348.

[eva70012-bib-0034] Mugniery, D. , O. Plantard , S. Fournet , et al. 2007. “Évaluation de L'efficacité et de la Durabilité des Résistances à *Globodera pallida* pa2/3, Provenant de *Solanum vernei*, *S. spegazzinii* et *S. sparsipilum* .” Nematologia Mediterranea 35, no. 2: 143–153.

[eva70012-bib-0035] Mwangi, J. M. , B. Niere , M. R. Finckh , S. Krüssel , and S. Kiewnick . 2019. “Reproduction and Life History Traits of a Resistance Breaking Population.” Journal of Nematology 51, no. 1: 1–13.

[eva70012-bib-0036] Ngala, B. , P. Dewaegeneire , E. Robilliard , et al. 2024. “Lure and Starve: Host Root Exudates to Suppress Field Populations of Cyst Nematodes.” Applied Soil Ecology 201: 105490.

[eva70012-bib-0037] Ngala, B. , N. Mariette , M. Ianszen , et al. 2021. “Hatching Induction of Cyst Nematodes in Bare Soils Drenched With Root Exudates Under Controlled Conditions.” Frontiers in Plant Science 11: 602825. 33488649 10.3389/fpls.2020.602825 PMC7820344

[eva70012-bib-0038] Nicol, J. M. , S. J. Turner , D. L. Coyne , L. D. Nijs , S. Hockland , and Z. T. Maafi . 2011. Current Nematode Threats to World Agriculture, 21–43. Dordrecht: Springer Netherlands.

[eva70012-bib-0039] Niere, B. , S. Krüssel , and K. Osmers . 2014. “Auftreten Einer Außergewöhnlich Virulenten Population der Kartoffelzystennematoden.” Journal für Kulturpflanzen 66, no. 12: 426–427.

[eva70012-bib-0040] Orlando, V. , and E. Boa . 2023. “Potato Cyst Nematodes: A Persistent and Fearsome Foe.” Plant Pathology 72, no. 9: 1541–1556.

[eva70012-bib-0041] Phillips, M. S. , C. A. Hackett , and D. L. Trudgill . 1991. “The Relationship Between the Initial and Final Population Densities of the Potato Cyst Nematode *Globodera pallida* for Partially Resistant Potatoes.” Journal of Applied Ecology 28, no. 1: 109–119.

[eva70012-bib-0042] Price, J. , K. Preedy , V. Young , D. Todd , and V. C. Blok . 2023. “Stacking Host Resistance Genes to Control *Globodera pallida* Populations With Different Virulence.” European Journal of Plant Pathology 168: 373–381.

[eva70012-bib-0043] Price, J. A. , D. Coyne , V. C. Blok , and J. T. Jones . 2021. “Potato Cyst Nematodes *Globodera rostochiensis* and *G. pallida* .” Molecular Plant Pathology 22, no. 5: 495–507. 33709540 10.1111/mpp.13047 PMC8035638

[eva70012-bib-0044] Robinson, M. P. , H. J. Atkinson , and R. N. Perry . 1987. “The Influence of Temperature on the Hatching, Activity and Lipid Utilization of Second Stage Juveniles of the Potato Cyst Nematodes *Globodera rostochiensis* and *G. pallida* .” Revue de Nématologie 10, no. 3: 349–354.

[eva70012-bib-0045] Saubin, M. , S. De Mita , X. Zhu , B. Sudret , and F. Halkett . 2021. “Impact of Ploidy and Pathogen Life Cycle on Resistance Durability.” Peer Community Journal 1: e8.

[eva70012-bib-0046] Schleker, A. S. S. , M. Rist , C. Matera , et al. 2022. “Mode of Action of Fluopyram in Plant‐Parasitic Nematodes.” Scientific Reports 12, no. 1: 11954. 35831379 10.1038/s41598-022-15782-7 PMC9279378

[eva70012-bib-0047] Scholte, K. 2000. “Screening of Non‐Tuber Bearing Solanaceae for Resistance to and Induction of Juvenile Hatch of Potato Cyst Nematodes and Their Potential for Trap Cropping.” Annals of Applied Biology 136, no. 3: 239–246.

[eva70012-bib-0048] Schouten, H. J. 1993. “Models of Incomplete Selection for Virulence of Potato Cyst Nematodes Caused by Sex Determination That Depends on Host Resistance.” Netherlands Journal of Plant Pathology 99: 191–200.

[eva70012-bib-0049] Schouten, H. J. 1994. “Preservation of Avirulence Genes of Potato Cyst Nematodes Through Environmental Sex Determination: A Model Involving Complete, Monogenic Resistance.” Phytopathology 84, no. 7: 771.

[eva70012-bib-0050] Schouten, H. J. 1996. “A Model Examining the Effect of Environmental Sex Determination in Parasites on the Breakdown of Monogenic Host Resistance.” Nematologica 42, no. 1: 80–88.

[eva70012-bib-0051] Shimizu, K. , R. Akiyama , Y. Okamura , et al. 2023. “Solanoeclepin B, a Hatching Factor for Potato Cyst Nematode.” Science Advances 9, no. 11: eadf4166. 36921046 10.1126/sciadv.adf4166 PMC10017031

[eva70012-bib-0052] Tobin, J. , P. Haydock , M. Hare , S. Woods , and D. Crump . 2008. “Effect of the Fungus *Pochonia chlamydosporia* and Fosthiazate on the Multiplication Rate of Potato Cyst Nematodes (*Globodera pallida* and *G. rostochiensis*) in Potato Crops Grown Under UK Field Conditions.” Biological Control 46, no. 2: 194–201.

[eva70012-bib-0053] Trudgill, D. , M. Phillips , and M. Elliott . 2014. “Dynamics and Management of the White Potato Cyst Nematode *Globodera pallida* in Commercial Potato Crops.” Annals of Applied Biology 164, no. 1: 18–34.

[eva70012-bib-0054] Turner, S. J. 1996. “Population Decline of Potato Cyst Nematodes (*Globodera rostochiensis*, *G. pallida*) in Field Soils in Northern Ireland.” Annals of Applied Biology 129, no. 2: 315–322.

[eva70012-bib-0055] Van Den Hoogen, J. , S. Geisen , D. Routh , et al. 2019. “Soil Nematode Abundance and Functional Group Composition at a Global Scale.” Nature 572, no. 7768: 194–198. 31341281 10.1038/s41586-019-1418-6

[eva70012-bib-0056] Varandas, R. , C. Egas , and I. L. Conceicao . 2020. “Potato Cyst Nematodes: New Solutions to an Old Problem.” Crop Protection 137: 105303.

[eva70012-bib-0057] Ward, S. A. , R. Rabbinge , and H. Den Ouden . 1985. “Construction and Preliminary Evaluation of a Simulation Model of the Population Dynamics of the Potato Cyst Nematode *Globodera pallida* .” Netherlands Journal of Plant Pathology 91: 27–44.

[eva70012-bib-0058] Zasada, I. A. , J. M. Halbrendt , N. Kokalis‐Burelle , J. LaMondia , M. V. McKenry , and J. W. Noling . 2010. “Managing Nematodes Without Methyl Bromide.” Annual Review of Phytopathology 48: 311–328. 10.1146/annurev-phyto-073009-114425 20455696

