# Supplementary material for: Combining Masculinizing Resistance, Rotation, and Biocontrol to Achieve Durable Suppression of the Potato Pale Cyst Nematode: A Model
Source: Evol Appl. 2024 Sep 19;17(9):e70012. doi: 10.1111/eva.70012 (PMC11411199; doi:10.1111/eva.70012)
Supplement: Supplementary file 1 — Data S1 [file EVA-17-e70012-s001.pdf]

# Supplementary Materials from "Combining masculinizing resistance, rotation and biocontrol to achieve durable suppression of the potato pale cyst nematode: a model"

Israël Tankam Chedjou, Josselin Montarry, Sylvain Fournet, Frédéric M. Hamelin

## Supplementary Material S1. Step-by-step modelling and analysis of the demo-genetic model with masculinizing resistance

### S1.1 Full model

The nematode population dynamics model is stage-structured. We also keep track of three genotypes in the nematode population: the avirulent genotypes $AA$ and $Aa$, and the virulent genotype $aa$.

The densities of encysted eggs (and juveniles J1) are denoted $E_{AA}$, $E_{Aa}$, and $E_{aa}$. The densities of free-living J2 juveniles, thereafter larvae, are $J_{AA}$, $J_{Aa}$, and $J_{aa}$. The densities of adult females are $F_{AA}$, $F_{Aa}$, and $F_{aa}$. The densities of adult males are $M_{AA}$, $M_{Aa}$, and $M_{aa}$.

Figure S3 shows how the stages occur over a growing season in the model. Growing seasons are indexed by $k \in \mathbb{N}$. The date the growing season $k+1$ begins is $t_k$. The duration of the larval stage is $\tau_1$. The duration of the adult stage is $\tau_2 - \tau_1$.

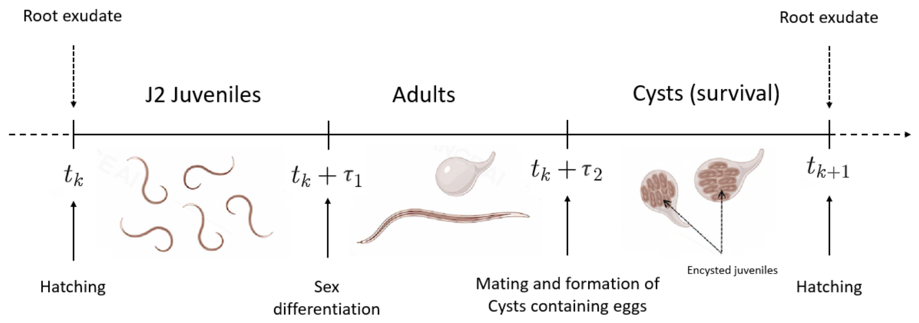


Figure S1: Schematic representation of how the stages occur over a growing season in the model.

The host is either susceptible or resistant, with two possible forms of resistance: blocking resistance, or masculinizing resistance. As indicated in the main text, since we assume virulence is initially present in the nematode population, the demographic dynamics are the same regardless of whether one grows a susceptible host or a blocking-resistance. From now on, we thus consider only two types of host for simplicity: a susceptible host or masculinizing resistance. The dynamics are described as follows.

1. After hatching (at time $t_k$), the larvae genotype densities are:

$$J_{AA}(t_k), J_{Aa}(t_k), J_{aa}(t_k)\,. \tag{1}$$

2. A fraction $s$ of larvae, regardless whether they are virulent (genotype $aa$) or avirulent (genotypes $Aa$ and $Aa$), survive until reaching the differentiation time ($t_k + \tau_1$).

Avirulent larvae (genotypes $AA$ and $Aa$) differentiate into adults with a proportion $m_A(k)$ of males, and a proportion $1 - m_A(k)$ of females. Virulent larvae (genotype $aa$) differentiate into adults with a proportion of $m_a(k)$ of males, and a proportion $1 - m_a(k)$ of females.

Sex allocation depends on host resistance during season $k$:

$$m_A(k) = \begin{cases} m, & \text{if the host is susceptible} \\ 1\,, & \text{otherwise (if resistance is masculinizing)} \end{cases}, \tag{2a}$$

$$m_a(k) = m\,, \tag{2b}$$

in which $0 < m < 1$ is the male fraction when masculinizing resistance does not occur or is broken. The transition from the larval stage to the adult stage is given by the following equation:

$$\begin{cases} F_{AA}(t_k + \tau_1) = s(1 - m_A(k))J_{AA}(t_k) \\ F_{Aa}(t_k + \tau_1) = s(1 - m_A(k))J_{Aa}(t_k) \\ F_{aa}(t_k + \tau_1) = s(1 - m_a(k))J_{aa}(t_k) \\ M_{AA}(t_k + \tau_1) = sm_A(k)J_{AA}(t_k) \\ M_{Aa}(t_k + \tau_1) = sm_A(k)J_{Aa}(t_k) \\ M_{aa}(t_k + \tau_1) = sm_a(k)J_{Aa}(t_k) \end{cases}. \tag{3}$$

3. We assume that all adults survive until mating occurs (time $t_k + \tau_2$). We assume that males and females are homogeneously mixed, and that mating occurs at random. The next three steps lead to the female-to-egg transition from females to eggs.

   (i) We first derive the frequencies of allele $A$ and $a$ in males and females. The variables $M = M_{AA} + M_{Aa} + M_{aa}$ and $F = F_{AA} + F_{Aa} + F_{aa}$ are the total densities of males and females, respectively. The frequency of allele $A$ in females, the frequency of allele $a$ in females, the frequency of allele $A$ in males, and the frequency of allele $a$ in males, are respectively:

$$p_f(k) = \frac{F_{AA}(t_k + \tau_1) + \frac{1}{2}F_{Aa}(t_k + \tau_1)}{F(t_k + \tau_1)}\,, \tag{4a}$$

$$q_f(k) = 1 - p_f(k)\,, \tag{4b}$$

$$p_m(k) = \frac{M_{AA}(t_k + \tau_1) + \frac{1}{2}M_{Aa}(t_k + \tau_1)}{M(t_k + \tau_1)}\,, \tag{4c}$$

$$q_m(k) = 1 - p_m(k)\,. \tag{4d}$$

   (ii) The allelic frequencies yield the following next-generation genotypic frequencies:

$$\begin{cases} f_{AA}(k) = p_f(k)p_m(k) \\ f_{Aa}(k) = q_f(k)p_m(k) + p_f(k)q_m(k) \\ f_{aa}(k) = q_m(k)q_f(k) \end{cases}. \tag{5}$$

(iii) Egg density is then obtained, for each genotype, as the product of the total female density, the average number of eggs per female, $e$, and the next-generation genotype frequency from equation (5):

$$\begin{cases} E_{AA}(t_k+\tau_2) = eF(t_k+\tau_1)f_{AA}(k) \\ E_{Aa}(t_k+\tau_2) = eF(t_k+\tau_1)f_{Aa}(k) \\ E_{aa}(t_k+\tau_2) = eF(t_k+\tau_1)f_{aa}(k) \end{cases} . \quad (6)$$

**Remark.** Equation (6) encompasses polyandry, since males have equal access to females.

4. A fraction $(1-\mu)(1-h)$ of eggs naturally survives a single period of host absence. This fraction is the product of the average fraction of viable eggs $(1-\mu)$ with the fraction of cysts that did not accidentally hatch $(1-h)$.

   In addition to mortality, a fraction $(1-b)$ of nematodes survives biocontrol application ($b$ is the biocontrol efficacy fraction). Not using biocontrol amounts to setting $b=0$.

   The rotation number $r$ is the number of years without growing potatoes. We assume that biocontrol is applied every year, regardless of whether potato is grown or not.

   As the potato growing season is relatively short on an annual scale (16 to 18 weeks), we assume, for simplicity, that annual cyst mortality is the same regardless of whether the host absence period is one year or less (one year minus the growing season).

   At the beginning of the next season, i.e. at time $t_{k+1}$, we assume that all eggs hatch from cysts. Therefore, the nematode generations do not overlap.

   The transition from eggs to larvae is thus given by:

$$\begin{cases} J_{AA}(t_{k+1}) = \big[(1-\mu)(1-h)(1-b)\big]^{r+1}E_{AA}(t_k+\tau_2) \\ J_{Aa}(t_{k+1}) = \big[(1-\mu)(1-h)(1-b)\big]^{r+1}E_{Aa}(t_k+\tau_2) \\ J_{aa}(t_{k+1}) = \big[(1-\mu)(1-h)(1-b)\big]^{r+1}E_{aa}(t_k+\tau_2) \end{cases} . \quad (7)$$

To simplify notations, we introduce the reproduction number $R'$, accounting for biocontrol and rotation:

$$R' = e\big[(1-\mu)(1-h)(1-b)\big]^{r+1}s\,. \quad (8)$$

We also introduce the female fractions

$$\begin{cases} f_A(k) = 1-m_A(k) \\ f_a(k) = 1-m_a(k) \end{cases} . \quad (9)$$

## S1.2 Compact model

Renaming the larvae genotype densities $J_{AA}(t_k)=X_k$, $J_{Aa}(t_k)=Y_k$, $J_{aa}(t_k)=Z_k$ for convenience, we summarize equations (3-7) in the following discrete-time dynamical system:

$$\begin{cases} X_{k+1} = R' \dfrac{m_A(k) f_A(k) \left(X_k + \frac{1}{2} Y_k\right)^2}{m_A(k)(X_k + Y_k) + m_a(k) Z_k} \\[2em] Y_{k+1} = R' \dfrac{m_A(k)\left(f_a(k) Z_k + \frac{1}{2} f_A(k) Y_k\right)\left(X_k + \frac{1}{2} Y_k\right) + f_A(k)\left(X_k + \frac{1}{2} Y_k\right)\left(m_a(k) Z_k + \frac{1}{2} m_A(k) Y_k\right)}{m_A(k)(X_k + Y_k) + m_a(k) Z_k} \\[2em] Z_{k+1} = R' \dfrac{\left(f_a(k) Z_k + \frac{1}{2} f_A(k) Y_k\right)\left(m_a(k) Z_k + \frac{1}{2} m_A(k) Y_k\right)}{m_A(k)(X_k + Y_k) + m_a(k) Z_k} \end{cases} . \quad (10)$$

We modify model (10) to account for intraspecific competition among larvae to host access, in a Beverton-Holt form:

$$\begin{cases} X_{k+1} = \dfrac{R'}{1 + c(X_k + Y_k + Z_k)} \dfrac{m_A(k) f_A(k) \left(X_k + \frac{1}{2} Y_k\right)^2}{m_A(k)(X_k + Y_k) + m_a(k) Z_k} \\[2em] Y_{k+1} = \dfrac{R'}{1 + c(X_k + Y_k + Z_k)} \dfrac{m_A(k)\left(f_a(k) Z_k + \frac{1}{2} f_A(k) Y_k\right)\left(X_k + \frac{1}{2} Y_k\right) + f_A(k)\left(X_k + \frac{1}{2} Y_k\right)\left(m_a(k) Z_k + \frac{1}{2} m_A(k) Y_k\right)}{m_A(k)(X_k + Y_k) + m_a(k) Z_k} \\[2em] Z_{k+1} = \dfrac{R'}{1 + c(X_k + Y_k + Z_k)} \dfrac{\left(f_a(k) Z_k + \frac{1}{2} f_A(k) Y_k\right)\left(m_a(k) Z_k + \frac{1}{2} m_A(k) Y_k\right)}{m_A(k)(X_k + Y_k) + m_a(k) Z_k} \end{cases} , \quad (11)$$

in which $c$ is an intraspecific competition parameter.

System (11) defines our demo-genetic model of potato cyst nematode population dynamics.

## S1.3 Basic demographic model

If only susceptible plant hosts are grown over years, the male fractions are $m_A(k) = m_a(k) = m$ and the female fractions are $f_A(k) = f_a(k) = (1 - m)$, for all $k \geq 0$. Model (11) simplifies as:

$$\begin{cases} X_{k+1} = \dfrac{(1-m)R'}{1 + c(X_k + Y_k + Z_k)} \dfrac{\left(X_k + \frac{1}{2} Y_k\right)^2}{X_k + Y_k + Z_k} \\[2em] Y_{k+1} = \dfrac{2(1-m)R'}{1 + c(X_k + Y_k + Z_k)} \dfrac{\left(Z_k + \frac{1}{2} Y_k\right)\left(X_k + \frac{1}{2} Y_k\right)}{X_k + Y_k + Z_k} \\[2em] Z_{k+1} = \dfrac{(1-m)R'}{1 + c(X_k + Y_k + Z_k)} \dfrac{\left(Z_k + \frac{1}{2} Y_k\right)^2}{X_k + Y_k + Z_k} \end{cases} . \quad (12)$$

Let $N_k = X_k + Y_k + Z_k$ be the total nematode density. System (12) yields:

$$N_{k+1} = (1-m)R' \frac{N_k}{1 + cN_k} . \quad (13)$$

This is the classical Beverton-Holt model. It has at most two non-negative equilibria: $N = 0$, and $N = ((1-m)R' - 1)/c$ (if and only if $R' > 1$). The nematode-free equilibrium, $N = 0$, is stable if and only if $(1-m)R' < 1$. Otherwise (if $(1-m)R' > 1$), the nematode population grows until reaching its carrying, $K(R', m, c) = ((1-m)R' - 1)/c$.

Next we show that genotype frequencies stabilize at the Hardy-Weinberg equilibrium. Using equations (12) and (13) yields the following genotype frequencies:

$$\begin{cases} f_{AA}(k) = \dfrac{X_{k+1}}{N_{k+1}} = \dfrac{\left(X_k + \frac{1}{2}Y_k\right)^2}{N_k^2} \\ \\ f_{Aa}(k) = \dfrac{Y_{k+1}}{N_{k+1}} = 2\dfrac{\left(Z_k + \frac{1}{2}Y_k\right)\left(X_k + \frac{1}{2}Y_k\right)}{N_k^2} \\ \\ f_{aa}(k) = \dfrac{Z_{k+1}}{N_{k+1}} = \dfrac{\left(Z_k + \frac{1}{2}Y_k\right)^2}{N_k^2} \end{cases}. \tag{14}$$

The frequency of allele $a$ on season $k$ is $a_k = (Z_k + \frac{1}{2}Y_k)/N_k$. Using equation (14), one can check that $a_{k+1}/a_k = 1$ for all $k \geq 0$. Therefore, $a_k = a_0$ for all $k > 0$. The Hardy-Weinberg equilibrium follows: for all $k > 0$, $f_{AA}(k) = (1 - a_0)^2$, $f_{Aa}(k) = 2a_0(1 - a_0)$, and $f_{aa}(k) = a_0^2$.

## S1.4 Demo-genetic model with masculinizing resistance

In this section, we consider masculinizing resistance to be the only potato variety grown over years. The male fractions are therefore $m_A(k) = 1$, $m_a(k) = m$ for all growing seasons $k \geq 0$; consequently, the female fractions are $f_A(k) = 0$ and $f_a(k) = (1 - m)$ for all $k \geq 0$.

Equation (11) yields $X_{k+1} = 0$ for all $k \geq 0$, meaning that the homozygous avirulent genotype $(AA)$ cannot persist. Therefore, $N_k = Y_k + Z_k$ for all $k > 0$. Equation (11) simplifies as:

$$\begin{cases} Y_{k+1} = (1 - m)R' \dfrac{Z_k}{1 + cN_k} \dfrac{\frac{1}{2}Y_k}{Y_k + mZ_k} \\ \\ Z_{k+1} = (1 - m)R' \dfrac{Z_k}{1 + cN_k} \dfrac{mZ_k + \frac{1}{2}Y_k}{Y_k + mZ_k} \end{cases}. \tag{15}$$

Summing both equations above yields:

$$N_{k+1} = (1 - m)R' \frac{Z_k}{1 + cN_k}.$$

Let the frequency of the virulent genotype $(aa)$ on season $k$ be

$$v_k = \frac{Z_k}{N_k}.$$

We obtain

$$v_{k+1} = \frac{Z_{k+1}}{N_{k+1}} = \frac{mZ_k + \frac{1}{2}Y_k}{mZ_k + Y_k} = \frac{mv_k + \frac{1}{2}(1 - v_k)}{mv_k + (1 - v_k)}$$

Model (15) can therefore be equivalently expressed as a demo-genetic model coupling population and virulence dynamics:

$$\begin{cases} N_{k+1} = (1 - m)R' \dfrac{N_k}{1 + cN_k} v_k \\ \\ v_{k+1} = \dfrac{mv_k + \frac{1}{2}(1 - v_k)}{mv_k + (1 - v_k)} \end{cases}. \tag{16}$$

## Non-dimensionalization

We rescale model (16) by letting $n_k = cN_k$, which yields:

$$\begin{cases} n_{k+1} = (1-m)R'\dfrac{n_k}{1+n_k}v_k \\ \\ v_{k+1} = \dfrac{mv_k + \frac{1}{2}(1-v_k)}{mv_k + (1-v_k)} \end{cases} . \tag{17}$$

## Equilibria

The fixed points of Equation (17) are $(0,1)$, $((1-m)R'-1,1)$, $(0,v^\star)$ and $(n^\star,v^\star)$, with

$$v^\star = \frac{1}{2(1-m)}\,, \tag{18}$$

and

$$n^\star = \frac{R'}{2} - 1\,. \tag{19}$$

## Stability of equilibria

The stability of a given fixed point $(\bar{n},\bar{v})$ depends on the spectral radius of the following matrix:

$$J(\bar{n},\bar{v}) = \begin{pmatrix} (1-m)R'\frac{1}{(1+n)^2}\bar{v} & (1-m)R'\frac{\bar{n}}{1+\bar{n}} \\ \\ 0 & \frac{m}{2\left(m\bar{v}+1-\bar{v}\right)^2} \end{pmatrix}.$$

- We have $J(0,1) = \begin{bmatrix} (1-m)\,R' & 0 \\ 0 & \frac{1}{2m} \end{bmatrix}$.

  The eigenvalues of $J(0,1)$ are $(1-m)R'$ and $\frac{1}{2m}$. Hence, the fixed point $(0,1)$ is stable iff $m \geq \frac{1}{2}$ and $(1-m)R' < 1$.

- We have $J((1-m)R'-1,1) = \begin{bmatrix} \frac{1}{(1-m)R'} & (1-m)\,R'-1 \\ 0 & \frac{1}{2m} \end{bmatrix}$.

  The eigenvalues of $J((1-m)R'-1,1)$ are $\frac{1}{(1-m)R'}$ and $\frac{1}{2m}$. Hence, the fixed point $((1-m)R'-1,1)$ is stable iff $m \geq \frac{1}{2}$ and $\frac{1}{(1-m)R'} < 1$, i.e. $(1-m)R' > 1$.

- We have $J(0,v^\star) = \begin{bmatrix} \frac{R'}{2} & 0 \\ 0 & 2m \end{bmatrix}$.

  The eigenvalues of $J(0,v^\star)$ are $\frac{R'}{2}$ and $2m$. Hence, the fixed point $(0,v^\star)$ is stable iff $m < \frac{1}{2}$ and $R' < 2$.

- We have $J(n^\star,v^\star) = \begin{bmatrix} \frac{2}{R'} & -(-1+m)(R'-2) \\ 0 & 2m \end{bmatrix}$.

The eigenvalues of $J(n^\star,v^\star)$ are $\frac{2}{R'}$ and $2m$. Hence, the fixed point $(n^\star,v^\star)$ is stable iff $m < \frac{1}{2}$ and $R' > 2$.

Table 1 summarizes the stability of the fixed points.

| Fixed Point | Density | Virulence | Stability | |
|---|---|---|---|---|
| | | | $m \geq .5$ | $m < .5$ |
| $(0,1)$ | Zero | Fixation | stable iff $(1-m)R' < 1$ | unstable |
| $((1-m)R'-1,1)$ | Maximum | Fixation | stable iff $(1-m)R' > 1$ | unstable |
| $(0,v^\star)$ | Zero | Polymorphism | unstable | stable iff $R' < 2$ |
| $(n^\star,v^\star)$ | Intermediate | Polymorphism | unstable | stable iff $R' > 2$ |

Table 1: Fixed points of the demo-genetic model (17) and their stability.

## S1.5 Time to effective suppression

The nematode pest can be considered as effectively suppressed from growing season $k^\dagger$ if its density, $N_k$, does not exceed a certain acceptance threshold, $\tau$, for all $k \geq k^\dagger$. We are interested in deriving the length of time required to achieve effective suppression, $k^\dagger(r+1)$. For simplicity, we consider that the frequency of the virulent genotype, $v_k$, is initially at equilibrium: i.e., for all $k \geq 0$, $v_k = v^\star$, as defined in equation (18). Model (16) simplifies as:

$$N_{k+1} = (1-m)R' \frac{N_k}{1+cN_k} v^\star \,. \tag{20}$$

We assume $N_0 > \tau$ (the nematode population density is initially above the acceptance threshold). We next focus on dynamics leading the pest to extinction, which occur if and only if $(1-m)Rv^\star < 1$, or equivalently $R < 2$. Using the explicit solution of equation (20), that is

$$N_k = \frac{\left(1-\frac{R}{2}\right) N_0}{\left(1-\frac{R}{2}+cN_0\right)\left(\frac{R}{2}\right)^{-k} - cN_0} \,,$$

we derive the generation $k^\dagger$ from which, for all $k \geq k^\dagger$, $N_k < \tau$: the latter inequality is equivalent to

$$\left(1-\frac{R}{2}\right) N_0 < \tau \left( \left(1-\frac{R}{2}+cN_0\right)\left(\frac{R}{2}\right)^{-k} - cN_0 \right) \iff \frac{\frac{\left(1-\frac{R}{2}\right)N_0}{\tau} + cN_0}{1-\frac{R}{2}+cN_0} < \left(\frac{R}{2}\right)^{-k} \,,$$

which can be equivalently expressed as

$$\log\left( \frac{\frac{\left(1-\frac{R}{2}\right)N_0}{\tau} + cN_0}{1-\frac{R}{2}+cN_0} \right) < -k \log\left(\frac{R}{2}\right) \iff k > \frac{\log\left( \frac{\frac{\left(1-\frac{R}{2}\right)N_0}{\tau} + cN_0}{1-\frac{R}{2}+cN_0} \right)}{-\log\left(\frac{R}{2}\right)} \,.$$

Hence,

$$k^\dagger = \left\lceil \log\left( \frac{\left(1-\frac{R}{2}\right)\frac{N_0}{\tau} + cN_0}{1-\frac{R}{2}+cN_0} \right) \log\left(\frac{2}{R}\right) \right\rceil \,. \tag{21}$$

# Supplementary Material S2. Additionnal simulations

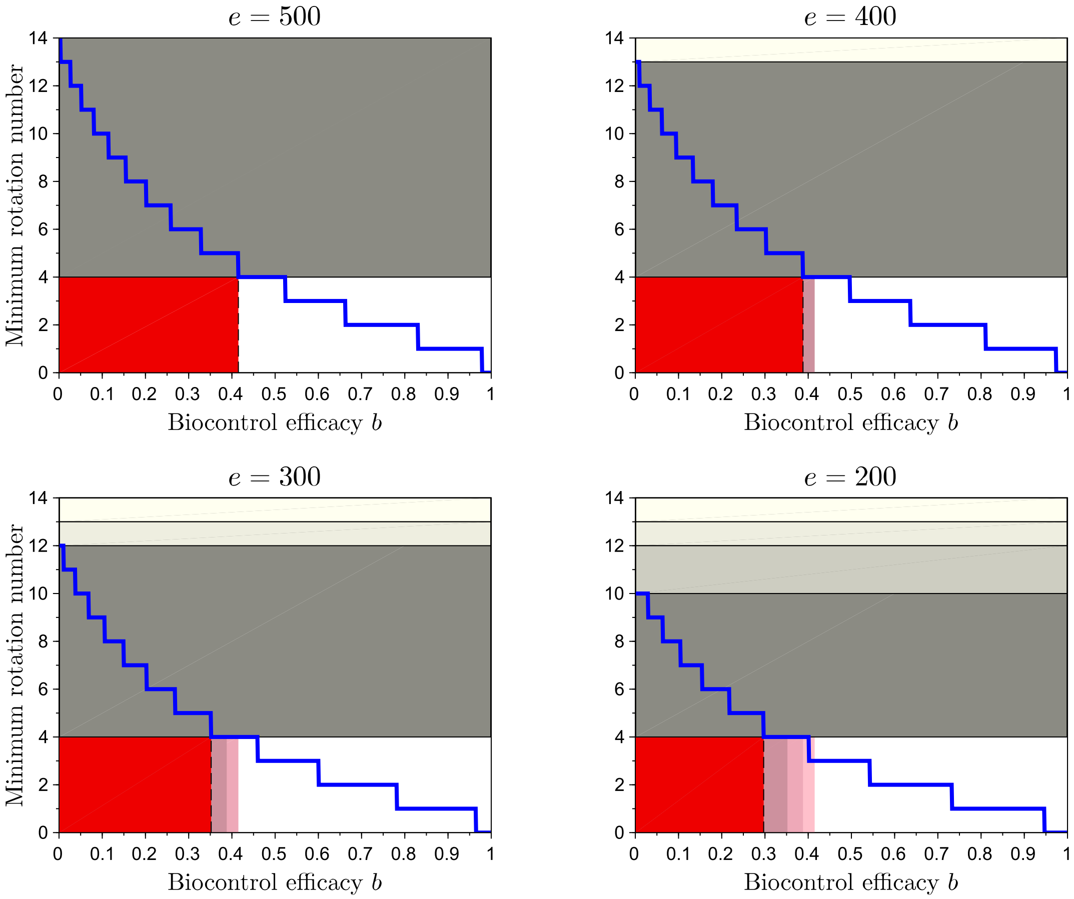


Figure S2: Rotation number required for long-term suppression of *G. pallida* under masculinizing resistance as a function of the biocontrol efficacy $b$ for different values of the number of eggs per cyst, $e$. The minimum rotation number is maximized for $b = 0$, and is a decreasing function of $e$. This graphic also illustrates the decrease of biocontrol efficacy needed as $e$ diminishes.

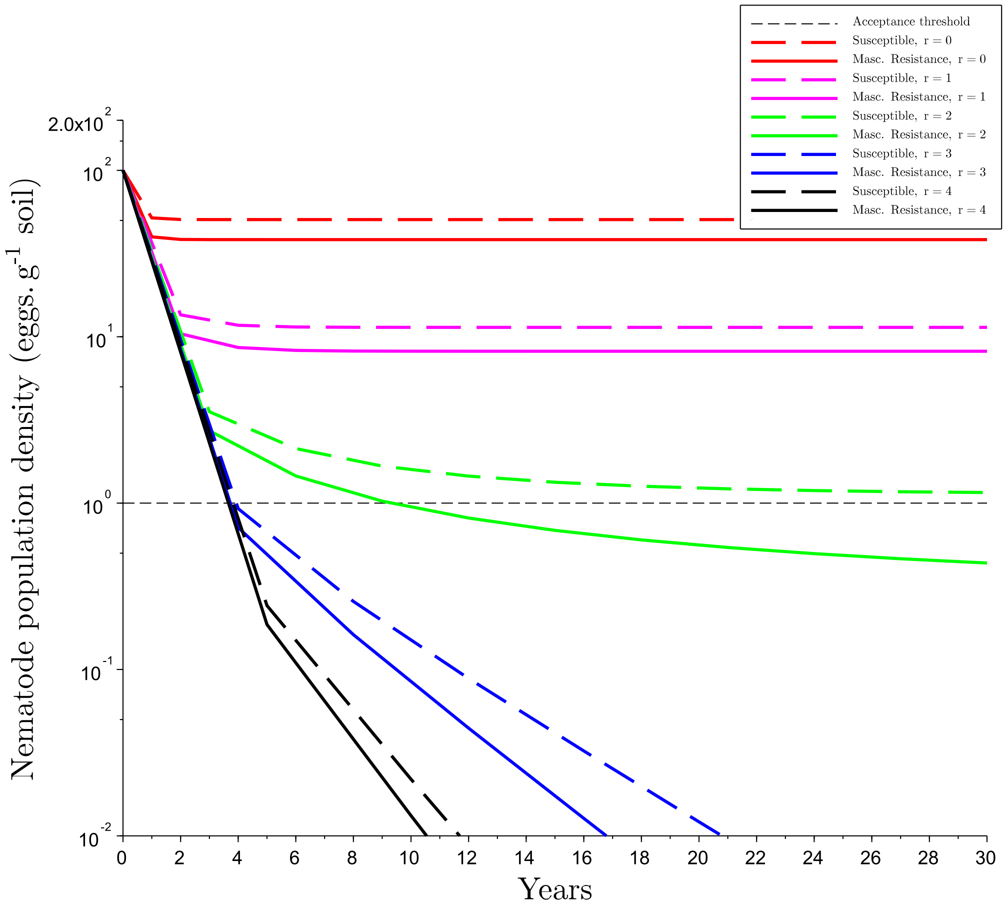


Figure S3: Time required to decrease nematode density under the acceptance threshold $\tau = 1$ nematode per gram of soil, with biocontrol efficacy fraction $b = .65$ and default parameter values, for a range of rotation numbers: $r = 0, 1, 2, 3, 4$. For high rotation numbers ($r = 3, 4$), the masculinizing resistance does not particularly speed up nematode suppression (initially at virulence frequency $v = v^{\star}$). By contrast, for $r = 2$, growing a susceptible variety does not achieve suppression, whereas masculinizing resistance does. Lower rotation numbers ($r = 0, 1$) do not achieve suppression.
